# Methanogenic response to long-term permafrost thaw is determined by paleoenvironment

**DOI:** 10.1093/femsec/fiaa021

**Published:** 2020-02-07

**Authors:** Stine Holm, Josefine Walz, Fabian Horn, Sizhong Yang, Mikhail N Grigoriev, Dirk Wagner, Christian Knoblauch, Susanne Liebner

**Affiliations:** 1 GFZ German Research Centre for Geosciences, Section Geomicrobiology, Telegrafenberg, 14473 Potsdam, Germany; 2 Universität Hamburg, Institute of Soil Science, 20146 Hamburg, Germany; 3 Universität Hamburg, Center for Earth System Research and Sustainability, 20146 Germany; 4 Russian Academy of Sciences, Siberian Branch, Melnikov Permafrost Institute, 677007 Yakutsk, Russia; 5 Potsdam University, Institute of Geosciences, 14476 Potsdam, Germany; 6 Potsdam University, Institute of Biochemistry and Biology, 14476 Potsdam, Germany

**Keywords:** Siberia, glacial and interglacial permafrost, methanogenic archaea, methane

## Abstract

Methane production in thawing permafrost can be substantial, yet often evolves after long lag phases or is even lacking. A central question is to which extent the production of methane after permafrost thaw is determined by the initial methanogenic community. We quantified the production of methane relative to carbon dioxide (CO_2_) and enumerated methanogenic (*mcrA*) gene copies in long-term (2–7 years) anoxic incubations at 4 °C using interglacial and glacial permafrost samples of Holocene and Pleistocene, including Eemian, origin. Changes in archaeal community composition were determined by sequencing of the archaeal 16S rRNA gene. Long-term thaw stimulated methanogenesis where methanogens initially dominated the archaeal community. Deposits of interstadial and interglacial (Eemian) origin, formed under higher temperatures and precipitation, displayed the greatest response to thaw. At the end of the incubations, a substantial shift in methanogenic community composition and a relative increase in hydrogenotrophic methanogens had occurred except for Eemian deposits in which a high abundance of potential acetoclastic methanogens were present. This study shows that only anaerobic CO_2_ production but not methane production correlates significantly with carbon and nitrogen content and that the methanogenic response to permafrost thaw is mainly constrained by the paleoenvironmental conditions during soil formation.

## Teaser

This study investigates the response of methane producing microorganisms in permafrost to long-term thaw lasting 2–7 years.

## INTRODUCTION

Up to about 1300 Pg of carbon is stored in northern terrestrial landscapes affected by permafrost, which is more carbon than currently in the atmosphere (Hugelius *et al*. 2014). Permafrost is defined as material frozen for more than two consecutive years (Brown and Kupsch 1974). Permafrost underlies a shallow layer of surface soil (the active layer), which thaws during the short summer season. Permafrost environments are often characterized by waterlogged soils as a result of restricted surface drainage caused by the underlying, permanently frozen ground. A 25% loss of the current permafrost area is predicted to occur in the next 100 years due to warming-induced thaw (Deng *et al*. 2014). The thawed organic matter may be mineralized to carbon dioxide (CO_2_) and methane (CH_4_) by enhanced microbial activity, thereby resulting in an increased release of greenhouse gases (GHG) into the atmosphere on a pan-Arctic scale (Wagner *et al*. 2005; Schuur *et al*. 2015; Knoblauch *et al*. 2018). The mobilization of carbon due to permafrost thaw, the associated release of GHG and their radiative forcing constitute the permafrost carbon feedback to climate change (PCF) (Schuur *et al*. 2015), which is predicted to cause global warming of up to 0.4°C by the year 2300 (Schneider von Deimling *et al*. 2015).

The microbial communities that drive the production of GHG in response to permafrost thaw are an important variable in the PCF (Graham *et al*. 2012). The process of methane production (methanogenesis) is suggested to be one of the most prominent microbial processes during the anoxic decomposition of organic matter in permafrost environments (Wagner *et al*. 2005; Koch, Knoblauch and Wagner 2009; Barbier *et al*. 2012; Graham *et al*. 2012; Knoblauch *et al*. 2018). Methanogenic archaea belonging to the phylum of Euryarchaeaota require anoxic conditions and are responsible for microbial methane production (Balch *et al*. 1979; Garcia, Patel and Olliver 2000; Wagner *et al*. 2013). Within the methanogenic Euryarchaeota, methane is produced by two major pathways: (i) the fermentation of acetate into methane and CO_2_ and (ii) the reduction of CO_2_ to methane coupled with the oxidation of formate or H_2_. All known methanogenic archaea express the methyl-coenzyme M reductase, which is involved in the final step of methanogenesis (Thauer 1998). The *mcrA* gene, which encodes the alpha subunit of the methyl-coenzyme M reductase, can therefore be used as a functional gene marker for methanogens. A homologue to the Euryarchaeota *mcrA* gene has recently been identified by metagenomics affiliated with the phylum Bathyarchaeota (Evans *et al*. 2015). However, final evidence for methanogenesis through Bathyarchaeota is still lacking (Evans *et al*. 2015). Recently, other phyla have been suggested to be capable of methane production based on genome reconstructions (Nobu *et al*. 2016; Vanwonterghem *et al*. 2016), but final proof of methane producing activity in these phyla remains lacking.

The ratio of CO_2_/CH_4_ produced under anoxic conditions over time indicates the extent to which the mineralization of organic matter occurs via methanogenesis. Quantifying the relative amount of carbon released as CO_2_ or methane is essential in determining biological feedback mechanisms between permafrost ecosystems and climate change since methane has a 28–34-time higher global warming potential (GWP) on a 100-year time horizon than CO_2_ (Myhre *et al*. 2013). CO_2_/CH_4_ ratios approaching 1 are indicative of fully methanogenic conditions (Thauer 1998). To date, GHG production in permafrost environments has mainly been assessed via active layer incubations (Kätterer *et al*. 1998; Ganzert *et al*. 2007; Coolen *et al*. 2011; Schädel *et al*. 2014; Chen *et al*. 2016; Yang *et al*. 2017; Faucherre *et al*. 2018) and only a few studies have focused on GHG production from older, ice-bonded deposits spanning the Holocene and Late Pleistocene (Wagner *et al*. 2007; Lee *et al*. 2012; Treat *et al*. 2015; Walz *et al*. 2018). These primarily short-term incubation studies allowed for important insights into immediate changes of overall methanogenic activity during permafrost thaw. Based on the CO_2_/CH_4_ ratios found in these studies, methane production from thawing permafrost has been assumed to play a minor role within the PCF compared with CO_2_ due to low production rates and long lag phases, which last from months to years (Waldrop *et al*. 2010; Coolen *et al*. 2011; Mackelprang *et al*. 2011; Knoblauch *et al*. 2013). Recently, methane production was shown to be capable of equaling CO_2_ production in long-term incubations in samples in which methanogenic archaea had initially been present (Knoblauch *et al*. 2018). This finding emphasizes the importance of linking methane production activity with molecular microbial analyses of archaeal community composition and abundance. A number of molecular ecological studies considered archaeal community structure and the abundance in permafrost (Wagner *et al*. 2007; Mackelprang *et al*. 2011; Graham *et al*. 2012; Hultman *et al*. 2015; Wei *et al*. 2018) and not just of the active layer (Wagner *et al*. 2005; Ganzert *et al*. 2007; Liebner *et al*. 2015) and proposed that the methanogenic community in ancient permafrost deposits reflects paleoclimatic conditions (Bischoff *et al*. 2013). However, changes in archaeal community structure and abundance in response to thaw have thus far been neglected, and studies that combine long-term anoxic incubations with archaeal community analysis remain lacking.

The aim of the present study is therefore to investigate how methane production rates and archaeal community structure and abundance change after long-term permafrost thaw. Furthermore, this study seeks to understand what determines this response. We address the following hypotheses: (i) The development of methane production after long-term permafrost thaw is determined by the initial methanogenic community size and composition and (ii) methanogenic abundance, community composition and diversity change substantially in response to long-term permafrost thaw. To address these hypotheses, we conducted long-term anoxic incubations on ancient permafrost spanning the Holocene to the last interglacial (Eemian) measuring the production of CO_2_ and methane in combination with a molecular microbial analysis of the archaeal community abundance, diversity and community composition.

## METHODS

### Study sites and sampling

**Figure 1.**
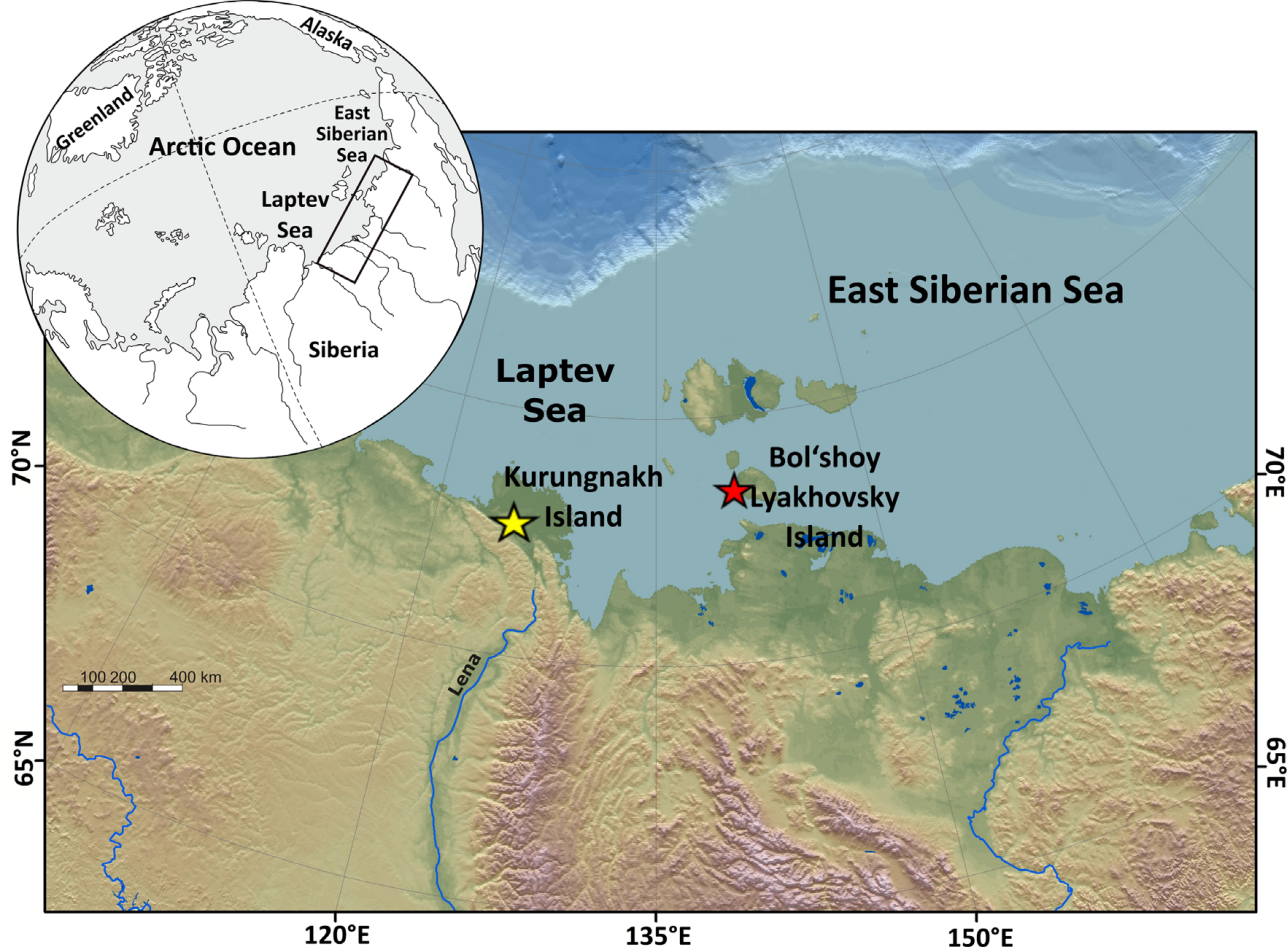
Location of the two study sites. Kurungnakh Island (72.33°N, 126.28°E) (yellow star) and Bol'shoy Lyakhovsky Island (73.35°N, 141.30°E) (red star). Modified from Wetterich *et al*. 2014.

We investigated samples obtained during two drilling campaigns in Northeast Siberia: one on Kurungnakh Island (72.33°N, 126.28°E) and the second on Bol'shoy Lyakhovsky Island (73.35°N, 141.30°E) (Fig. 1). In July 2002, during the Russian–German LENA expedition, a 25-meter-deep core was drilled on Kurungnakh Island. Drilling took place on a low-centered ice wedge polygon at the shore of the Lena River (Bischoff *et al*. 2013; Knoblauch *et al*. 2013). In April 2014, four drill cores were obtained from the southern coast on Bol'shoy Lyakhovsky Island (Table 1) as part of the Russian–German research project CARBOPERM (Schwamborn and Schirrmeister 2014). For Bol'shoy Lyakhovsky Island, a composite core was constructed based on previous stratigraphic studies and age assignment (Schirrmeister et al. 2002, 2011; Andrev et al. 2004, 2009; Wetterich et al. 2009, 2014; Zimmerman *et al*. 2017).

**Table 1.** Age, Marine Isotope Stage (MIS), core sample ID, properties of the paleoenvironment, location (site) and coordinates of all permafrost deposits investigated. MIS and the assigned age period for Bol'shoy Lyakhovsky Island samples were based on Andreev *et al*. (2004, 2009), on Wetterich *et al*. (2009, 2014) and Zimmerman *et al*. (2017). For Kurungnakh Island, the age assignment was based on Knoblauch *et al*. (2013, 2018). The information on paleoenvironmental conditions was derived from Schirrmeister *et al*. (2002) (1); Andreev *et al*. (2009) (2) , Grosse *et al*. (2007) (3). Sher *et al*. (2005) (4), Schirrmeister *et al*. (2011) (5), Wetterich *et al*. (2014) (6) and Wetterich *et al*. (2008) (7). The table was modified from Stapel *et al*. (2018). For information on core ID or replicate ID, see SI Table 1.

Age Epoch	Regional chrono-stratigraphy	Time interval	MIS	Core ID	Sample ID	Paleoenvironment	Site	Drilling coordinates
Holocene	Holocene	Interglacial	1 <11 ka BP	L_0502	L_0502.1 L_0502.2	Climate warming (1) and permafrost thawing (2), moisture increase, thermokarst formation (2), unstable environmental conditions (2)	Bol'shoy Lyakhovsky Island	73.34996°N 141.24156°E
Pleistocene	Sartan	Glacial (Stadial)	2 14–29 ka BP	K_1356	K_1356.1 K_1356.2	Cold and dry conditions (7)	Kurungnakh Island	72.333°N 126.283°E
				K_1358	K_1358.1 K_1358.2			
				K_1359	K_1359.1 K_1359.2			
	Kargin	Glacial (Interstadial)	3 34–42 ka BP			Rising temperature and precipitation (7)		
			3.1	K_1362	K_1362.1 K_1362.2			
				K_1363	K_1363.1 K_1363.2			
				K_1364	K_1364.1 K_1364.2			
		Glacial (Interstadial)		L_0508	L_0508.1 L_0508.2	Rising temperature and soil moisture (4), high organic matter accumulation (5), warmer summers, open vegetation (2)	Bol'shoy Lyakhovsky Island	73.34996°N 141.24156°E
		Glacial (Interstadial)	3.2	L_0203	L_0203.1 L_0203.2	Rising temperature and soil moisture (4), high organic matter accumulation (5), Warmer summers, open vegetation (2)		73.33623°N 141.32761°E
		Glacial (Interstadial)	3.3	L_0210	L_0210.1 L_0210.2	Warmer summers, open vegetation (2). Last interstadial optimum, warm and dry (6)		
	Zyryan	Glacial (Stadial)	4 40–52 ka BP	L_0315	L_0315.1 L_0315.2	Cold and dry climate (6), harsh climate conditions (2), thin snow cover, low precipitation (2)		73.33464°N 141.32822°E
	Eemian Kazansevo	Interglacial	5e ∼136 ka BP	L_0404	L_0404.1 L_0404.2	Warmer climate, open grass tundra similar to modern conditions (2), permafrost thawing (2), up to 4–5 °C higher summer temperatures than today, shrub tundra (2)		73.34100°N 141.28587°E
				L_0405	L_0405.1 L_0405.2			
				L_0407	L_0407.1 L_0407.2			

In both campaigns, drilling was performed without drilling fluids to avoid microbiological contamination of the permafrost samples, as drilling fluid may transport microorganisms from the active layer to the permafrost layers and penetrate into the permafrost core (Bang-Andreasen *et al*. 2017). Even though mixing of the permafrost sediments is assumed to be excluded due to frozen state of the core material, some mixing can still happen as drilling itself produces heat which creates drilling mud of layers above the actual drilling spot (Bang-Andreasen *et al*. 2017). The individual core segments, which were up to 50 cm in length, were placed into sterile plastic bags immediately after removal from the corer and stored at about −8 °C. The cores were transported in frozen conditions in insulated containers with cool packs to Potsdam, Germany following Wagner *et al*. (2007). All permafrost material was kept frozen at about −20 °C until further processing. Core segments were split along their axis into halves using sterile sampling devices, with a diamond saw in an ice laboratory at −10 °C. During subsampling the outer core layer (1–2 cm) was removed in order to remove potentially contaminated outer soil layers (Bang-Andreasen *et al*. 2017). Subsamples for the different analysis were filled into sterile plastic Nalgene boxes.

The permafrost deposits formed under a timeinterval within an ice age are defined as glacial permafrost. The glacial permafrost is further divided in stadials (Marine Isotope Stage (MIS) 2, 4) and interstadials (MIS 3.1, 3.2, 3.3). Thus, stadials are periods of colder climate while interstadials are periods of warmer climate. Permafrost deposits formed between ice ages were formed in periods of warmer climate and are defined as interglacial deposits (MIS 1 and 5e).

Kurungnakh Island belongs to the oldest terrace of the Lena Delta, which was formed during the Middle to Late Pleistocene. The terrace consists of fine-grained silty sediments with a high content of segregated ice, massive ice wedges and an organic carbon content between 2 and 12% (Wetterich *et al*. 2008). During the Holocene and Late Pleistocene, Kurungnakh Island underwent several climatic changes, which were identified by paleoclimatic and environmental reconstruction (Wetterich *et al*. 2008). Based on a combination of fossil bioindicators, such as pollen, plant macrofossils, insect remains, mammal bones and ^14^C radiocarbon dating (Wetterich *et al*. 2014), the Late Pleistocene deposits were divided into a cold and dry unit (3–14 m, Late Weichselian, MIS 2, Sartan stadial, with an age of 14–29 ka BP and into a warmer and wetter unit (14–22 m, Middle Weichselian, 32–42.5 ka BP, MIS 3, Kargin interstadial) (Wetterich *et al*. 2014) (Table 1). In this study, deposits from depths of 3−22 m, which spanned the Sartan stadial (MIS 2 and the Kargin interstadial (MIS 3) (Wetterich *et al*. 2008), were studied. The four drilling sites of Bol'shoy Lyakhovsky (Table 1) were chosen to maximize the stratigraphic coverage and time span. In this study, we used samples from the Holocene (MIS 1), the Sartan stadial (MIS 2), the Kargin interstadial (MIS 3), the Zyryan stadial (MIS 4) and the Eemian interglacial (MIS 5e). More information on the drilling locations and sample properties is given in Stapel *et al*. (2018). Stadial deposits were formed during an extreme cold and dry climate, while the climate of the interstadials was similar to that of today (Laukhin *et al*. 2006; Wetterich *et al*. 2008).

### Incubation and gas measurement

Anoxic incubations that were used to determine CO_2_ and methane production rates were established according to Knoblauch *et al*. (2018). This means that triplicates of 20 g of sediment for each sample were placed in 120-mL bottles and closed with butyl rubber stoppers. The samples were handled under a constant flow of molecular nitrogen to minimize oxygen exposure. Each of the triplicates was amended with 5 mL oxygen free water and the headspace was repeatedly exchanged with pure nitrogen. Enough molecular nitrogen was added to establish a slight overpressure inside each bottle to ensure that oxygen did not enter the bottle. In occasional cases of negative pressure differences between headspace pressure and ambient pressure, 5–10 mL of molecular nitrogen was added to reestablish overpressure. The samples were incubated in the dark at 4 °C for 2501 days (Kurungnakh Island) and for up to 1229 days (Bol'shoy Lyakhovsky Island), respectively. Carbon dioxide and methane concentrations in the headspace were measured with a gas chromatograph (7890, Agilent Technologies, USA) that was equipped with a nickel catalyst to reduce CO_2_ to methane and with a flame ionizing detector (FID). The gases were separated on a PorapakQ column with helium as a carrier gas (Walz *et al*. 2018). The amount of gas in the headspace was calculated based on headspace volume, incubation temperature and pressure inside the bottle using the ideal gas law. The amount of gas dissolved in water was calculated based on the gas concentration in the headspace, pressure inside the bottle, water content, pH and gas solubility. Solubility for CO_2_ and methane in water at 4 °C was calculated according to Carroll, Slupsky and Mather (1991) and Yamamoto *et al*. (1976), respectively. To account for the dissociation of carbonic acid in water at different pH values, dissociation constants from Millero *et al*. (2007) were applied. For this study, we included representative duplicates out of the triplicates for further molecular analysis. This also means that the presentation of CO_2_ and methane production rates refers to the duplicates chosen for molecular analysis. The detection limit for methane production was 50 nmol CH_4_ gdw^-1^ (Knoblauch *et al*. 2018).

### DNA extraction and amplicon sequencing

Extraction of total DNA was optimized by using two different kits based on yield and quality. DNA was extracted from ∼0.5 g of soil per sample using the Genematrix DNA extraction kit (Roboklon, EurX) and Fastspin (MP Biomedicals). Mostly, the Roboklon kit (Roboklon, EurX) was used, however in samples where the Roboklon failed to extract DNA the Fastspin (MP Biomedicals) was used. Spin columns from Fastspin were consistently applied and all samples were homogenized with the Fastprep-24 from MP Biomedicals. As mentioned above, DNA of the incubated samples was extracted from representative duplicates (one DNA extraction per duplicate) out of the triplicates. For the initial material (prior to incubation), two DNA extractions per sample were done. The DNA concentration was checked on a Qubit 2.0 (Invitrogen). The replicates were sequenced separately.

The archaeal 16S rRNA genes were amplified using the primers 20F and 958R (100 μM) (Delong 1992) with an Optitaq polymerase (Roboklon, Germany) in a concentration of 1.25 U. A template volume of 5 μL and a total of 25 μL of reaction volume were used. Annealing was performed at 55°C and a total of 40 amplification cycles were used. Initially we checked for positive archaeal amplification after 40 cycles, which was very rare. It was therefore decided to apply a standardized method of the nested PCR for every sample according to Winkel *et al*. (2018). The second PCR was performed with the primers Arch349R (10 μM) and Arch806-R (10 μM) (Takai and Horikoshi 2000). Three microliters of the first PCR reaction was used as a template. A reaction volume of 50 μL was used and annealing again took place at 55°C. The second PCR had 35 amplification cycles. The PCR amplification was carried out with a T100 Thermal Cycler (Bio-Rad Laboratories, CA, USA). For each sample, a unique combination of barcodes was used. The PCR products were purified using Agencourt Ampure Xp (Agencourt Bioscience, USA) by applying 50 μL of PCR product and 180 μL of magnetic bead solution. Sequencing was done by Eurofins Scientific (Konstanz, Germany) using the Illumina Miseq v3 platform (Illumina Inc., San Diego, CA, USA) and Illumina Miseq v3 kits (2 x 300 bp). In addition, 15% PhiX virus was used to account for low diversity in the samples. We included positive and negative controls during the PCR runs, in order to assess the potential contamination and PCR artifacts which might occur during the high number of cycles used. We used sterile PCR water as template for the negative PCR control and DNA from a pure culture of the methanogenic archaeon *Methanosarcina Barkeri* as template for the positive control. In addition, we added two negative DNA extraction controls, with the negative DNA extract as template for the PCR. A negative DNA extraction control both for the genematrix DNA extraction kit (Roboklon, EurX) and one for the Fastspin (MP Biomedicals) were included. Despite including several controls (see results), we cannot exclude the introduction of artifacts during the PCR cycles potentially resulting in an over- or underestimation of certain archaeal taxa. The controls were sequenced and analyzed together with all other samples.

### Sequence analysis and bioinformatics

Libraries were demultiplexed using Cutadapt (Martin 2011) where primer sequences had a maximum error rate of 10% and barcodes were required to have a phred score higher than Q25 without any mismatches. Samples were further processed using the DADA2 pipeline (Callahan *et al*. 2016). Read sequences were truncated (250/200 forward/reverse) and quality-filtered before the error model was generated. After dereplication, sample inference and merging of the paired-end reads, reads are required to have a minimum length of 200 bp. The orientation of the reads was standardized by calculating the hamming distances of the sequences and their reverse complement. The sequence table has been created and chimeras were removed using a de novo approach. The amplicon sequence variants (ASV) were assigned to the SILVA taxonomy database (v132) (Quast *et al*. 2013) using vsearch (Rognes *et al*. 2016) as utilized in the framework of QIIME2 (Bolyen *et al*.^2019^). Sequences were assigned to taxonomy based on a 99% sequence similarity threshold. Relative abundances are used for the visualization and comparison of the microbial composition of the samples in order to account for different sequencing depths. Raw sequencing data is available at the European Nucleotide Archive (ENA) under BioProject accession number PRJEB33214 and sample accession number ERS3543634-ERS3543676.

### Quantification of *mcrA* gene copy numbers

The quantification of the functional *mcrA* gene copy number was performed as described in Yang *et al*. (2017) using the CFX Connect Real-Time PCR Detection System (Bio-Rad Laboratories, Inc., Hercules, USA) and the primer set mlas-F/mcrA-R (Hales *et al*. 1996). Each reaction (20 μL) contained two-times concentrate of KAPA HiFi SYBR Green (KAPA Biosystems), 100 μM of the forward and reverse primer, sterile water and 4 μL of DNA template. The environmental DNA samples were diluted 10–100 fold and each run included technical triplicates. An annealing temperature of 60 °C was used. Melt curve analysis from 65 to 95 °C with a 0.5 °C-temperature increment per 0.5 s cycle was conducted at the end of each run to identify nonspecific amplification of DNA. Genomic standards of *Methanosarcina barkeri* were included in each qPCR that was run to ensure both linearity and expected slope values of the Ct/log curves. PCR efficiency was calculated based on the standard curve using the BioRad CFX. The efficiency varied between 80% and 100%, with an R^2^ value of 0.98–0.99. Abundances of the *mcrA* gene were calculated per gram of dry weight (dw) of soil. The detection limit for gene copies was 100 gene copies per gram dry weight of the soil.

### Statistics

Absolute read counts were transformed to relative abundances to standardize the data and to account for different sequencing depths. All ASVs not assigned to Archaea on highest taxonomic level were removed prior to statistical analysis. Variation in ASV composition between samples and the determination of diversity were assessed using the Past 3.12 software (Hammer, Harper and Ryan 2001). Diversity indices were calculated based on the total counts of all ASVs including unassigned, only with the exception of Bacterial ASVs. For the initial samples (prior to incubation), we present diversity values as calculated means from the two DNA extractions, while for the incubated samples we present diversity values independently for the two replicates. The significance of change in Shannon diversity index was tested by a student's *t-*test. The taxonomic abundance across samples was visualized through a bubble plot generated in R by the ggplot2 v.3.1.0 package (Wickham 2016). For the creation of the bubble plot presentation unassigned ASVs and the ASVs assigned to Bacteria on the highest taxonomic level have been removed. The relative abundance of the ASVs are presented at genus level. Taxa which could not be assigned down to genus level are present at a higher taxonomic level. To visualize the grouping patterns of permafrost deposits based on paleoclimate, environment and age, a nonmetric multidimensional scaling (NMDS) based on the Bray–Curtis dissimilarity was used (Past 3.12 software, Hammer, Harper and Ryan 2001). The significance of the shift in community composition was tested by a one way Permanova test, applying the Bray Curtis dissimilarity. Mean values and standard error of the means were used to describe the obtained results of *mcrA* gene copies per gram dry weight. The correlation between maximum CO_2_ and CH_4_ and total organic carbon (TOC) and nitrogen (N) was analyzed by means of linear regression and described by Pearson's correlation coefficient.

## RESULTS

### Methane production relative to carbon dioxide production

**Figure 2.**
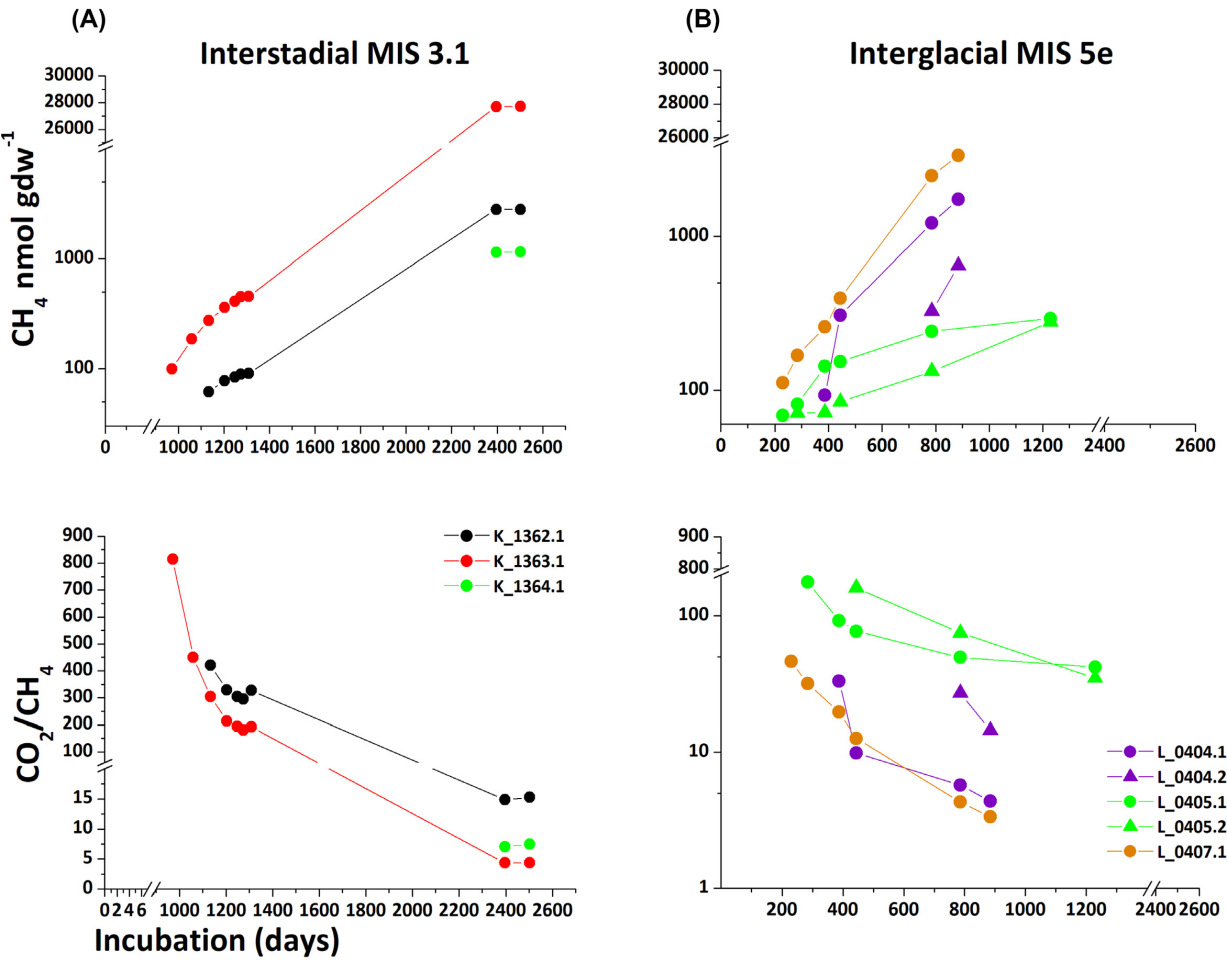
Cumulative methane production (nmol CH_4_ gdw^-1^) and ratios of CO_2_ and methane over the incubation period (days). Panel A and B present deposits with active methanogenesis; A) interstadial MIS 3.1 and B) interglacial, Eemian, MIS 5e. Methane production after a threshold of 50 nmol CH_4_ gdw^-1^ is presented.

Maximum methane production (nmol/gdw of soil) within the incubations (2–7 years) varied between 3.36 × 10^3^ and 2.77 × 10^4 ^nmol CH_4_ gdw ^-1^ of soil (Fig. 2). Methane production was observed in deposits of interstadial (MIS 3.1) and of interglacial Eemian (MIS 5e) origin. Methane production for MIS 1, 2, 3.2, 3.3 and 4 was below the detection limit of 50   nmol CH _4_ gdw ^-1^ of soil until the end of the experiment. The time period before methane production began- defined as the lag phase- lasted 230–2395 days and was shortest in the Eemian deposits (230–387 days). In these deposits, methane production occurred in all three samples investigated and reached 3.36 × 10^4 ^nmol CH_4_ gdw ^-1^ of soil after 885 days. Within the same incubation period, methane production of the interstadial deposits (MIS 3.1) was below the detection limit. In interstadial deposits of MIS 3.1, methane production was observed in 3 of 4 deposits and reached 1.15 × 10^3^– 2.77 × 10^4^ nmol CH_4_ gdw ^-1^ of soil after 2395 days. In addition to cumulative methane amounts shown in Fig. 2, methane production rates are presented in SI Figure S3. For the interstadial MIS 3.1 deposits an increase in methane production rate per day was observed from day 970 until day ∼1250 when a rate of 1.24 nmol of CH_4_ gdw ^-1^ d^-1^ was reached. After day 1250, methane production rates decreased approaching zero by the end of the incubation. For the interglacial Eemian deposits (MIS 5e) an increase in methane production rates was observed until the end of the incubation, reaching a maximum rate of 6.56 nmol CH_4_ gdw ^-1^ d ^-1^.

Anoxic production of CO_2_ was observed in all deposits. In general, the onset of methanogenesis was associated with a rise in CO_2_ production (SI Figure S2, SI Figure S4). The highest CO_2_ production was observed in the stadial deposits (MIS 2) reaching 6.02 × 10^4^ nmol CO_2_ gdw^-1^ and in the interstadial deposits (MIS 3.1) reaching 1.20 × 10^5 ^nmol CO_2_ gdw^-1^ after 2500 d. At the beginning of the incubation, very high CO_2_/CH_4_ ratios of up to ∼800 were observed due to very low methane production (Fig. 2). At the end of the incubations, a greater contribution of methanogenesis was observed in all samples with measurable methanogenesis (MIS 5e and MIS 3.1) and CO_2_/CH_4_ ratios decreased to 4. An up to 10-fold higher anoxic CO_2_ production was observed in samples from Kurungnakh Island compared with those from Bol'shoy Lyakhovsky Island independent of permafrost organic matter age. Maximum values of produced methane and CO_2_ in each deposit were correlated with total organic carbon (TOC) and total nitrogen content (N) using a Pearson correlation test (SI Table 8). Unlike methane production, the CO_2_ production correlated positively with increasing TOC (r=0.65) and N (r=0.79). All p-values for the Pearson correlation are presented in SI Table 8b.

### Methanogenic abundance initially and after long-term thaw

The interstadial MIS 3.1 and the Eemian deposits MIS 5e, displayed the highest initial abundance of methanogenic archaea of up to 8.9 × 10^7^ (lowest 5.6 × 10^3^) gene copy numbers gdw^-1^ (Figure SI 1 A). In MIS 2, initial methanogenic copy numbers varied between 9.38 × 10^5^– 2.2 × 10^6^ gene copies gdw^-1^. Initial *mcrA* gene copy numbers were lowest in Holocene deposits of MIS 1 ranging between 1.58 × 10^3^–2.53 × 10^3^ gene copies gdw^-1^ . In deposits from MIS 3.2, 3.3 and 4, which showed no methanogenic activity, the *mcrA* gene copies in the initial samples were below the detection limit of 100 gene copies gdw^-1^.

After long-term thaw, methanogenic archaea displayed the highest abundance in deposits in which methane production occurred, which is in interstadial deposits of MIS 3.1 (1.27 × 10^4^ – 6.11 × 10^5^ gene copies gdw^-1^) and in the Eemian deposits MIS 5e (1.76 × 10^3^ – 4.86 × 10^6^ gene copies gdw^-1^). Furthermore, methanogenic archaea were detected in the Holocene samples from MIS 1 (2.11 × 10^3^– 1.67 × 10^3^ gene copies gdw^-1^) and in interstadial samples (MIS 3.2 and MIS 3.3) from Bol'shoy Lyakhovsky (2.38 × 10^2^– 1.47 × 10^3^ gene copies gdw^-1^) in low abundance. Overall, no increase in methanogenic abundance was observed after long-term thaw except for the Eemian deposits, for which the abundance increased from 1.76 × 10^3^ - 2.87 × 10^3^ gene copies gdw^-1^ to 4.86 × 10^6^ gene copies gdw^-1^. The primers applied (mlas-F/mcrA-R) do also amplify the closely related *mrtA* gene, and the gene copies might therefore be overestimated up to a factor of two when both genes are present (Steinberg and Regan 2009). However, as the quantification of methanogenic archaea in this study is used to compare abundance between samples, this potential overestimation does not affect the derived conclusions.

### Archaeal community structure initially and after long-term thaw

A total of 1061 archaeal ASVs were identified. The maximum read length was 421 bp and the average length of all reads was 378 bp. The read counts versus the number of ASVs did not show any correlation (SI Figure S5). This confirms that an increasing number of reads does not correlate with increasing alpha diversity. The species richness as a function of sampling was assessed by a rarefaction curve (SI Figure S6). All curves reach plateaus and thereby an appropriate sequencing depth across all samples is assumed. Therefore, a bias due to sequencing depth can be ruled out. Positive and negative controls were sequenced and analyzed together with all other samples. The negative PCR control consisted of 1 read which was filtered away due to the low quality of the read. The negative DNA extraction controls consisted of one from the Fastspin (MP Biomedicals) with nine unassigned reads and one from the genematrix DNA extraction kit (Roboklon, EurX) with one unassigned read. The positive control consisted of 25967 reads with 100% assigned to *Methanosarcina* (SI Figure S8).

**Figure 3.**
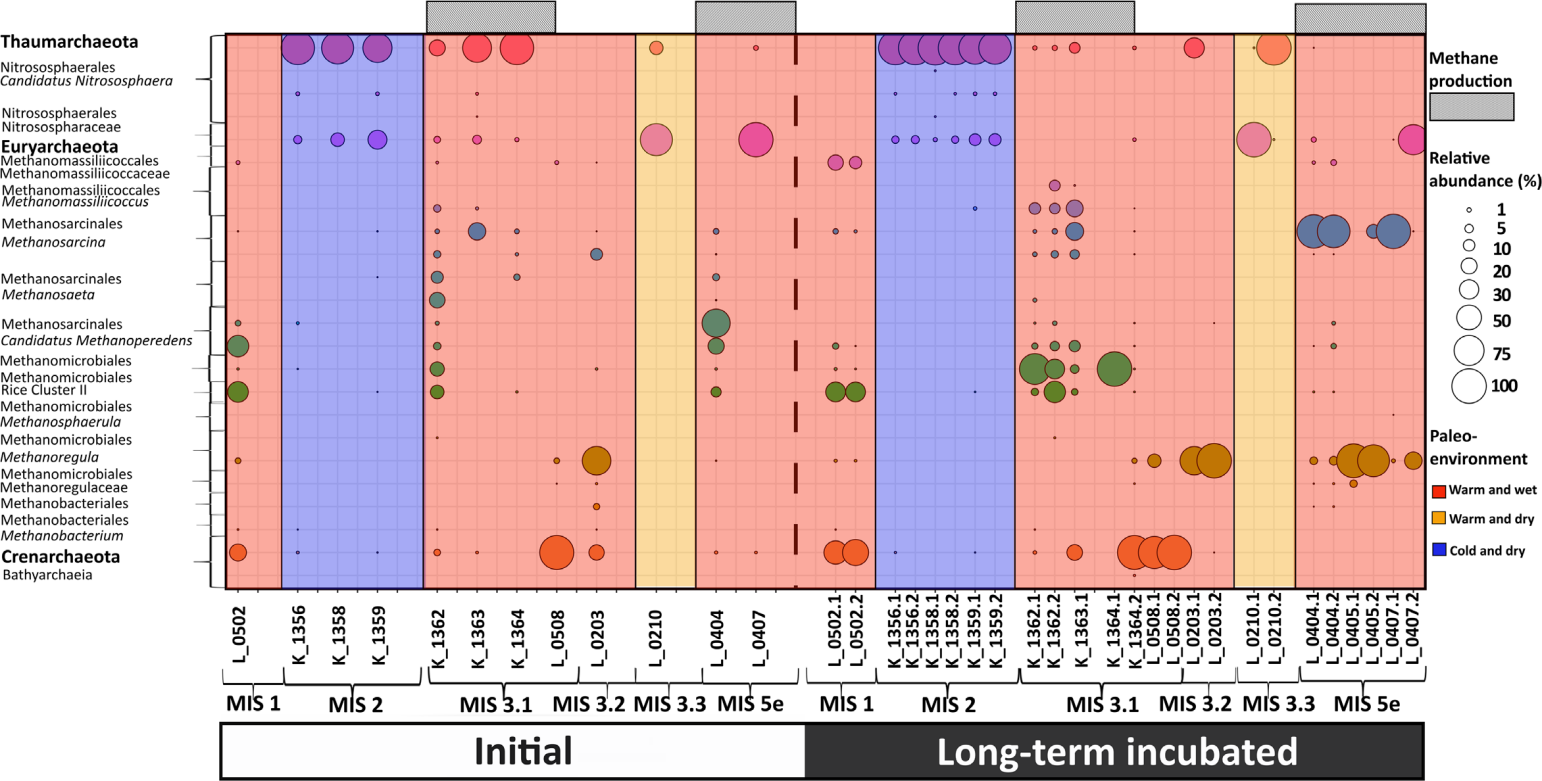
Archaeal community composition initially and at the end of the incubations. ASVs from biological initial replicates are merged. The lowest taxonomic assignment is presented down to the genus level. The samples are shown on the *x*-axis for all graphs, with samples from Bol'shoy Lyakhovsky Island marked with ‘L’ and those from the Kurungankh Island marked with ‘K.’ Only one incubation was available from the sample of MIS 3.1 (K_1363). The sample ID and replicate number (according to Table 1) are indicated in the legend of each panel. The blue fill color indicates samples that were formed under colder and drier and conditions (MIS 2). The red fill color indicates samples that were formed under periods with higher temperatures and rates of precipitation (MIS 1, 3.1, 3.2, 5e). The orange fill color indicates samples which were formed during warm albeit dry conditions (MIS 3.3).

The initial archaeal community and the community after long-term thaw are depicted in Fig. 3, in which the initial samples are merged and the incubated samples are presented by their individual replicates as described above. In a few cases, one of the replicates had reads below 1500 (SI Table S3−S7). In these cases, the merging was verified by comparing the community composition of the replicates. Data from all initial replicates are presented in SI Figure S7. In samples showing methane production during the incubations, a shift in the archaeal community structure and a decreasing trend in alpha diversity were observed via the Shannon diversity index at the end of the incubations (SI Figure S1B). Distinct differences in the initial archaeal community compositions were observed between stadial, interstadial and interglacial deposits. The stadial deposits that had formed under cold and dry conditions (MIS 2) were dominated by the Thaumarcheota of the order Nitrososphaerales (99–100% relative abundance). Methanogens were initially found to make up 0.03–0.7% of the total archaeal community and were only detected in one sample after long term thaw (K_1359.1, 0.7%). The deposits that were formed during the last interstadial optimum (MIS 3.3, L0210) under warmer albeit drier conditions were also initially found to be dominated by Thaumarchaeota of the order Nitrososphaeres. No change in community structure was observed by the end of the incubation in deposits of MIS 2 and MIS 3.3 (Fig. 3).

Interstadial and interglacial deposits that had formed under conditions with higher temperatures and precipitation (MIS 1, 3.1, 3.2 and 5e) were dominated by Euryarchaeota (46–99%), whereof the methanogenic archaea made up 22–98%. The initial archaeal community in Holocene (MIS 1) deposits was dominated by methanogenic orders of Methanosarcinales (52–53%) and Methanomicrobiales (47–48%). Within the order of Methanosarcinales, the putative anaerobic methanotrophic *Candidatus Methanoperedens* (80–100%) was dominating together with *Methanosarcina* (0–29%). Within the order of Methanomicrobiales, an uncultured archaeon dominated (90–94%) together with *Methanoregula* (6–10%). In addition, 23–25% of all archaeal reads were assigned to Bathyarchaeota. At the end of the incubations, a shift in community composition within the class of Methanomicrobia had occurred, leading to a dominance of Methanomicrobiales (88–99%). Furthermore, the order of Methanomassiliccocales within the Thermoplasmata had increased in abundance from 0.5–0.8% of all archaea initially to 12–18% by the end of the incubations. Bathyarchaeota had increased in abundance from 23–25% of all archaea to 45–56% by the end of the incubations.

In interstadial (MIS 3) samples, different communities were observed between the deposits from Kurungnakh Island and Bol'shoy Lyakhovsky Island. The initial archaeal community from Kurungnakh was dominated by the methanogenic orders Methanosarcinales (51–72%) and Methanomicrobiales (28–63%). Within the order of Methanosarcinales the families *Methanosaetaceae* and *Methanoperedenaceae* were dominating. Within Methanomicrobiales and Methanomassiliicoccales, the Rice cluster II and the genus *Methanomassiliicoccus* were dominating respectively. By the end of the incubation, a dominance of Methanomicrobiales of Rice cluster II and *Methanomassiliicoccus* were in general observed, except in one sample of MIS 3.1, (K_1363), in which *Methanosarcina* dominated after thaw. Deposits of interglacial MIS 1 and Interstadial MIS 3.1, with a dominance of Methanomicrobiales after thaw were in general associated with an increase in Methanomassiliccocales.

In the interstadial deposits (MIS 3.1) from Bol'shoy Lyakhovsky Island, the initial archaeal community was highly dominated by Bathyarchaeota (95–99%). No major shifts in community composition occurred after the incubation period. The interstadial MIS 3.2 samples of Bol'shoy Lyahovsky (L_0203) were initially dominated by Methanomicrobiales (72–79%) and Methanosarcinales (20%), with a high abundance of *Methanoregula* and *Methanosarcina* respectively. After long-term thaw, *Methanoregula* dominated (70–100%). The Eemian deposits of MIS 5e were initially highly dominated by *Methanoperedens* related ASVs within the order of Methanosarcinales (92–93%). At the end of the incubation, a shift in the community composition of these samples within the order of Methanosarcinales towards a dominance of *Methanosarcina* was observed. Furthermore, *Methanoregula* was found to be abundant by the end of the incubation period.

**Figure 4.**
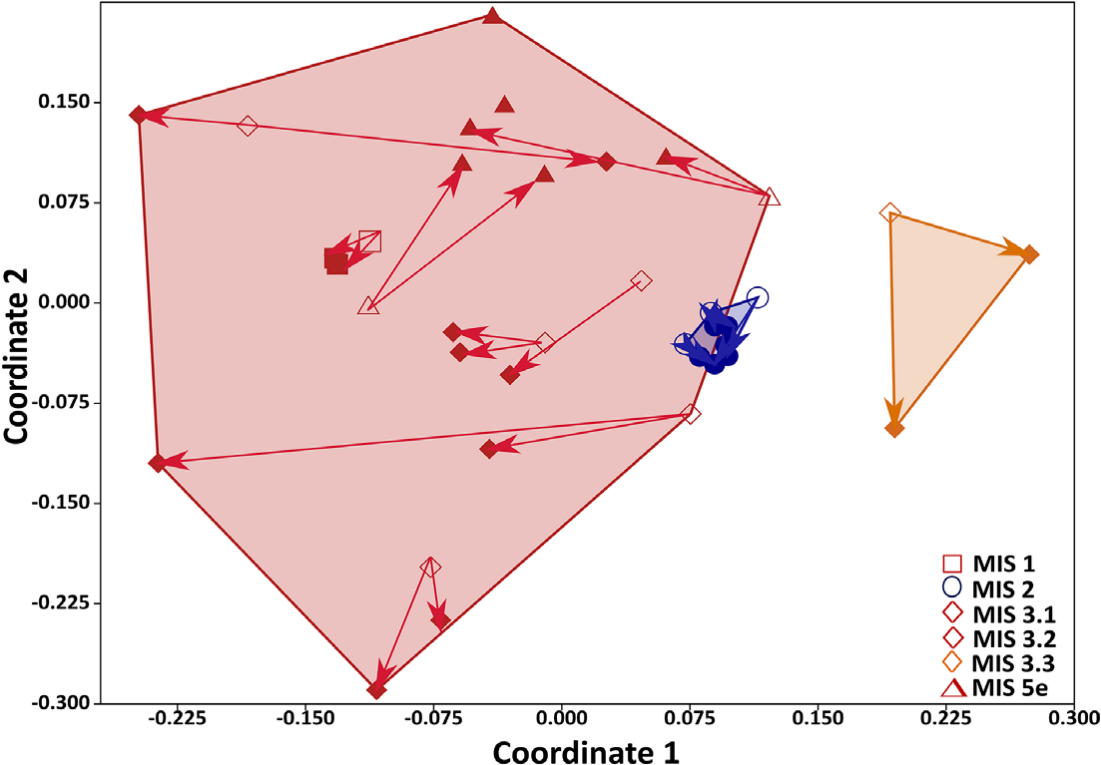
NMDS plot of the archaeal community composition based on Bray Curtis dissimilarity of initial and long-term incubated samples. The symbols depict the age of the deposits; □ MIS 1, ○ MIS 2, ◊ MIS 3.1, 3.2, ◊ MIS 3.3, ∆ MIS 5e. The stress value of NMDS was 0.32. Samples from interstadials and interglacials are marked with a red background, and samples from stadials are marked with a blue background. Samples from the last interstadial optimum formed during warm albeit dry conditions are marked with an orange background. The shifts in archaeal community composition between the initial samples and the long-term incubated samples are marked with arrows. The initial material of one deposit from MIS 5e (L_0405) were not available.

A NMDS ordination plot based on Bray−Curtis dissimilatory revealed a distinct clustering of samples originating from cold and dry conditions and samples that had formed under warmer and wetter conditions (Fig. 4). The corresponding grouping was thereby based on the paleoenvironmental conditions. A large shift in total archaeal community structure occurred during the incubations in the samples originating from soil that had formed under warmer and wetter conditions (MIS 1, 3.1, 3.2 and 5e), while a minor shift was observed in the samples originating from soil that had formed under cold and dry conditions (MIS 2 and MIS 3.3) and which showed no methanogenesis. The shifts in microbial community composition between the initial and long- term incubated samples were tested by a one-way Permanova test. The initial samples originating from cold and dry conditions (MIS 2 and MIS 3.3) and the initial samples originating from warm and wet conditions (MIS 1, 3.1, 3.2 and 5e) showed no significant difference in community composition, due to the presence of methanogenic archaea within two initial deposits of MIS 2. This can be observed in the NMDS ordination plot (Fig. 4) by the overlapping areas including deposits formed during warm and wet conditions and deposits formed during cold and dry conditions. Between the initial and long-term incubated samples originating from cold and dry conditions (MIS 2 and MIS 3.3) no significant shift was observed. Between initial and long-term incubated samples originating from warm and wet conditions (MIS 1, 3.1, 3.2 and 5e) no significant difference in community composition was observed, due to the divergence of the shifts. Long-term incubated samples that were formed under warm and wet (MIS 3.1, 3.2 and 5e) conditions and those formed under cold and dry conditions (MIS 2 and MIS 3.3) showed a significant difference in community composition (*P* = 0.0006). All p-values from the Permanova tests are presented in the supplementary material (SI Table S9).

## DISCUSSION

### Drivers of methane production following permafrost thaw

After thaw, methane production occurred in interstadial deposits of MIS 3.1 and in interglacial deposits of the MIS 5e (Eemian), where methanogenic archaea had initially been present, while a failed detection of methanogens coincided with a lack of methane production, even after up to seven years of incubation. These observations support earlier findings that methane production in thawing permafrost is constrained by the presence of methanogenic archaea (Knoblauch *et al*. 2018). Paleoclimatic conditions during permafrost aggradation that had initially been favorable for the establishment of methanogenic archaea hence determine the initial methane production after the thawing of ancient permafrost. The abundance of methanogens in this study is similar to previous enumerations of methanogens in permafrost (Rivkina *et al*. 1998) from the northeastern region of the Russian Arctic, and our measured methane production rates are in the range of the few previously reported long-term methane production rates from thawing permafrost (Rivkina and Gilichinsky 1996).

Despite this overall trend, some exceptions occurred. The samples from the Holocene did not produce methane even though methanogens were initially present, however in very low abundance. It is possible that for the Holocene-derived samples of this study the incubation period of 885 days was still insufficient since permafrost samples with lag phases for methane production of up to 2000 days had previously been observed (Knoblauch *et al*. 2018). Overall, the lack of methane production in the Holocene sample of this study appears to be exceptional because several related studies have documented methane production from Holocene deposits (Rivkina and Gilichinsky 1996; Ganzert *et al*. 2007; Wagner *et al*. 2007; Knoblauch *et al*. 2018). Another reason for inactive methanogenic communities might be that the studied Holocene deposit represents a drained thermokarst depression (Andreev *et al*. 2009; Stapel *et al*. 2018). It is likely that the thermokarst depression was exposed to oxic conditions while it drained causing unfavorable conditions for methanogens. This is supported by findings of van Huissteden *et al*. (2011), who observed a correlation between permafrost thaw lakes and drastically decreasing methane production. The lack of methane production despite the presence of *mcrA* genes in the Holocene samples is therefore likely a result of targeting dead cells. Moreover, significant *mcrA* gene copy numbers occurred in samples deriving from MIS 2 (Sartan stadial) characterized by cold and dry climatic conditions. According to paleoenvironmental reconstructions (Wetterich *et al*. 2009), the MIS 2 deposits sampled in this study experienced massive post-depositional erosion and disturbances that may have introduced allochthonous methanogenic archaea into these deposits. This hypothesis would explain the low abundance of methanogenic archaea within the total archaeal community in MIS 2 samples, which were instead dominated by Thaumarchaeota of the order Nitrososphaerales (99–100%). The deposits from MIS 3.2 and MIS 3.3 originated from the Middle Pleistocene and were formed under warmer temperature (Wetterich *et al*. 2011; Stapel *et al*. 2018). MIS 3.3 does however not contain a high proportion of methanogens as observed in MIS 3.2 and is instead also dominated by Thaumarchaeota. MIS 3.3 was formed during the last interstadial optimum, which consisted of warmer albeit drier conditions than MIS 3.1 and MIS 3.2 (Wetterich *et al*. 2011; Stapel *et al*. 2018). The dry paleoconditions might indicate aerobic soil conditions and thereby explain the lack of methanogenesis and the striking dominance of Thaumarchaeota in the deposits of MIS 3.3.

Freeze-locked methanogenic communities have previously been found to reflect the paleoenvironment (Bischoff *et al*. 2013; Rivkina *et al*. 2016; Vishinivetskaya *et al*. 2018). In greater detail, Bischoff *et al*. (2013) assigned warm and wet conditions during soil formation in flooded lowlands to suitable anoxic conditions for the growth and activity of methanogenic archaea. Our results now suggest that periods with higher temperatures and precipitation at the time of deposition also determine methanogenesis after long-term permafrost thaw. This finding is supported by shorter-term incubation studies on Late Pleistocene deposits from MIS 3, in which sediment deposited under wet conditions produced high amounts of greenhouse gases (Bishoff *et al*. 2013; Walz *et al*. 2018).

The oldest permafrost deposits investigated in the current study originated from the Eemian interglacial and revealed the highest methane production within an incubation period of 885 days. Additionally, elevated methane production occurred in samples originating from the Late Pleistocene compared with samples from younger Pleistocene deposits, which supports previous findings in which permafrost age did not correlate with methane production after thaw (Knoblauch *et al*. 2013). However, our finding that methane production occurs in ∼130.000 year old Eemian deposits, contradicts a study of a chronosequence in which increasing age was found to negatively coincide with the presence of methane pathways (Mackelprang *et al*. 2011). Methane production from the Eemian deposits, which were about two orders of magnitude higher than in the other studied samples, was comparable to previously determined methane production rates from Holocene deposits (Rivkina and Gilichinsky 1996; Ganzert *et al*. 2007; Wagner *et al*. 2007). Furthermore, methane production rates from the Eemian deposits were shown to increase until the end of the incubation, which indicate a potential for even higher methane production if the incubations would have continued. Furthermore, the high activity of the Eemian deposits was likely affected by their depositional environment, which was described as a lake environment (Andrev et al 2004, 2009; Wetterich *et al*. 2009). With the onset of the new ice age after the Eemian, the lake sediments may have got frozen without a period of drought. It is therefore reasonable to assume that the depositional environment during the Eemian initially created suitable conditions for methanogenesis (Schirrmeister *et al*. 2002). Aside from high methane production rates, the Eemian deposits also reached low CO_2_/CH_4_ ratios approaching 4:1, which indicates that a larger proportion of anoxic mineralization occurred via methanogenesis in the Eemian samples than in those of the other epochs.

The methane production after permafrost thaw was not only correlated with the presence of methanogens. Moreover, the archaeal communities of incubations showing methane production were dominated by methanogenic archaea, which constituted between 40 and 100% of all detected archaea. In contrast, the archaeal communities in permafrost deposits originating from soils formed under cold and dry conditions were dominated by Thaumarchaeota (99–100%). This coincides with previous findings in which Thaumarchaeota were found to dominate in oxic, nutrient-poor environments (Schleper 2010). Even though it cannot be excluded that Thaumarchaeota contain taxa which also thrive under anoxic condition.

Unlike the production of methane, CO_2_ production correlated significantly with total carbon and nitrogen content (SI Table S8a and S8b) which were not necessarily higher in deposits from warmer and wetter conditions than from colder and drier conditions. This also means that in comparison with CO_2_ production, carbon and nitrogen content were less important drivers for methanogenesis than paleoenvironmental conditions. This finding is consistent with previous work (Knoblauch *et al*. 2013; Schädel *et al*. 2014) and can be explained by the restriction of methanogenesis to a functionally narrow group of archaea, whereas anoxic CO_2_ can be produced by a highly diverse group of microorganisms.

### The response of the methanogenic community to long-term thaw

The response of methanogens to thaw was manifested by shifts in the methanogenic community composition and a drop in diversity rather than by a substantial increase in the number of methanogens. This finding is supported by previous work reporting on substantial changes in the methanogenic community structure after thaw (Hodgkins *et al*. 2014; McCalley *et al*. 2014; Liebner *et al*. 2015; Wei *et al*. 2018) but contradicts other studies that have observed no significant change (Prater, Chanton and Whiting 2007; Yang *et al*. 2017). In our study, we find a positive correlation between methane production and a warmer and wetter environment during deposition. The methane production was also found to correlate with a large shift in methanogenic community composition after the incubation.

Overall, we observed a shift in the methanogenic community towards a dominance of methanogens of the orders of Methanosarcinales and Methanomicrobiales at the end of the incubation. This finding suggests that these taxa contain members that are well adapted to the conditions after long term incubation such as substrate depletion. This shift occurred independently of the initially different methanogenic communities between the two study sites, which likely reflect regional environmental controls, such as differences in salinity, temperature and pH (Wen *et al*. 2017).

In the Holocene deposits of MIS 1, a shift within the class of Methanomicrobia occurred at the end of the incubation towards a dominance of Methanomicrobiales (88–99% of all Methanomicrobia). Methanomicrobiales consist solely of hydrogenotrophic methanogens. Furthermore, the order of Methanomassiliccocales increased in abundance from initially 0.5–0.8% of all archaea to 12–18% by the end of the incubations. The order of Methanomassiliicoccales is phylogenetically different from all other methanogens (Söllinger *et al*. 2015) and it describes the 7th order of methanogens. It was previously identified in Arctic wetlands (Winkel et al 2018). Methanomassiliicoccales lack the pathway for CO_2_ reduction to methyl co-enzyme M, and gain energy by a hydrogen dependent reduction of methanol or methylamines. Even though we cannot finally conclude that hydrogenotrophic methanogenesis is mainly taking place, the observed dominance of solely hydrogen dependent methanogens suggest that hydrogen driven systems are important after thaw. Liebner *et al*. (2015) observed a shift in the methanogenic pathway to H_2_/CO_2_ as main substrates along with permafrost thaw. This was supported by previous incubation studies of active-layer material reporting that the amendment of hydrogen leads to much higher methane production than the amendment of other substrates (Wagner *et al*. 2005; Ganzert *et al*. 2007). A similar community composition like in MIS 1 deposits was observed in deposits of Kurungnakh Island and Bol'shoy Lyakhovsky originating from MIS 3.1 (K_1364) and MIS 3.2 (L_0203). The process of methanogenesis is fueled by the establishment of fermentation processes, which is supported by the synchronized increase in both CO_2_ and methane production (SI Figure 4).

The high abundance of potentially hydrogenotrophic methanogens after thaw contradicts other studies that suggested that acetate is more important as a substrate in cold environments (Chin and Conrad 1995; Wagner and Pfeiffer 1997). While mostly hydrogenotrophic methanogens had increased by the end of the incubation, the Eemian deposits showed a dominance of *Methanosarcina*-affiliated taxa after thaw. Members of the genus *Methanosarcina* have a broad substrate spectrum including acetate, methanol and methylated compounds (Welte 2018), and they are estimated to be responsible for about two-thirds of the global biogenic methane production (Ferry and Lessner 2008). An adaptation of methanogens to experimental warming via taxonomic shifts toward acetoclastic methanogenesis was previously observed in permafrost-affected environments (Tveit *et al*. 2015). Recently, a high expression of transcripts involved in acetogenesis and acetoclastic methanogenesis has been found in thawing permafrost (Coolen and Orsi 2015; Wei *et al*. 2018). It is thus possible that the high methanogenic activity we observed in the Eemian deposits, which are dominated by *Methanosarcina* at the end of the incubations is due to acetoclastic methanogenesis.

In addition to the described shift in the methanogenic community structure, a decrease in diversity could generally be observed in deposits in which methanogenic archaea dominated. This observation might be expected due to stable laboratory conditions during the incubation and may reflect competitive advantages of individual methanogenic taxa among the initial methanogenic community. However, it may also point toward hampered recolonization during thaw due to laboratory batch incubation. Other studies that have studied natural landscape gradients reported an enhanced colonization by new organisms following permafrost thaw (McCalley *et al*. 2014; Hultman *et al*. 2015). Our observation that methanogenic archaea did not increase in abundance after thaw even when substantial methane production occurred contradicts findings of related studies, which have shown correlations between methanogenesis and methanogen abundance in permafrost-affected soils (Høj, Olsen and Torsvik 2005; Wagner *et al*. 2005; Waldrop *et al*. 2010; Lipson *et al*. 2013; Deng *et al*. 2014). Mackelprang *et al*. (2017) classified only 18–22% of the cells in permafrost as being alive, which points to a high proportion of inactive cells in the initial deposits. Thus, the decay of external DNA or of DNA from inactive cells and the growth of active taxa could have occurred simultaneously while still revealing similar *mcrA* copy numbers at the beginning and at the end of the incubation. We assume that there was an increase in active methanogenic cells and in the activity per cell. Nevertheless, substantial growth of the active methanogenic population in our incubations would still have led to a log-fold increase in *mcrA* copy numbers, which we did not observe.

## CONCLUSION

We observed that the long-term response of the methanogenic community is determined by the paleoclimate during soil formation. Soil that had formed during periods with increased temperatures and precipitation was found to preserve methanogenic communities and responded with substantial methane production to thawing conditions. Long-term thaw was also found to be associated with a substantial shift within methanogenic community composition, which was accompanied by a relative increase in hydrogenotrophic methanogens. Only the oldest permafrost deposits originating from the Eemian were found to be highly dominated by potential acetoclastic methanogens after long-term thaw. Since no correlation between carbon concentrations and methane production could be found, the projection of methane production from thawing permafrost based on soil carbon seems an oversimplification.

## Supplementary Material

fiaa021_Supplemental_Files
