# Supplementary material for: Methanogenic response to long-term permafrost thaw is determined by paleoenvironment
Source: FEMS Microbiol Ecol. 2020 Feb 7;96(3):fiaa021. doi: 10.1093/femsec/fiaa021 (PMC7046019; doi:10.1093/femsec/fiaa021)
Supplement: fiaa021_Supplemental_Files [file fiaa021_supplemental_files.zip › Figure_legends_Supporting_information.docx]

**Figure and Table legends for Supporting Information for:**

**The methanogenic response to permafrost thaw is determined by the paleoenvironment**

Stine Holm^1^, Josefine Walz^2,3^, Fabian Horn^1^, Sizhong Yang^1^, Mikhail N. Grigoriev^6^, Dirk Wagner^1,4^, Christian Knoblauch^2,3^, Susanne Liebner^1,5^

^1^GFZ German Research Centre for Geosciences, Section Geomicrobiology, Telegrafenberg, 14473 Potsdam, Germany, ^2^Universität Hamburg, Institute of Soil Science, 20146 Hamburg, Germany, ^3^Center for Earth System Research and Sustainability, Universität Hamburg, 20146 Germany, ^4^Universität Potsdam, Institute of Geosciences, 14476 Potsdam, Germany, ^5^Universität Potsdam, Institute of Biochemistry and Biology, 14476 Potsdam, Germany, ^6^Melnikov Permafrost Institute, Siberian Branch, Russian Academy of Sciences, Yakutsk, Russia

Correspondence: S. Liebner, Section Geomicrobiology, GFZ German Research Centre for Geosciences, Helmholtz Centre Potsdam, Telegrafenberg, Potsdam, Germany.

E-mail: sliebner@gfz-potsdam.de

**This SI file contains:**

**SI Figure 1*.*** *mcrA* gene copies and alpha diversity (Shannon H index)

**SI Figure 2.** CO_2_ production over time (nmol CO_2_ gdw^-1^)

**SI Figure 3.** CH_4_ production in rates (nmol CH_4_ gdw^-1^ d^-1^)

**SI Figure 4.** CH_4_ production (nmol CH_4_ gdw^-1^) and CO_2_ (nmol CH_4_ gdw^-1^) in samples with active methanogenesis.

**SI Figure 5.** Number of reads versus number of Amplicon Sequence Variants (ASVs)

**SI Figure 6.** Rarefaction curve showing sample size versus number of species.

**SI Figure 7.** Bubble plot presenting all replicates of initial samples

**SI Figure 8.** Krona charts of controls.

**SI Table 1.** Original core ID, replicates and sample ID of all samples included

**SI Table 2.** Methane production over time

**SI Table 3-7.** Reads per sample, number of archaeal ASVs, number of bacterial ASVs, unassigned.

**SI Table 8a+b.**Correlation between total organic carbon (TOC %) and nitrogen (%), and the maximum CO_2_ and CH_4_ values_._

**SI Table 9.** One-way Permanova test of shifts in community composition between initial and long term incubated samples.

**SI Figure 1*.* A)** Initial *mcrA* gene copies gdw^-1^ of soil and **B)** Alpha diversity (Shannon H). The blue fill color indicates samples that were formed under colder and drier and conditions (MIS 2). The red fill color indicates samples that were formed under periods with higher temperatures and rates of precipitation (MIS 1, 3.1, 3.2, 5e). The orange fill color indicates samples which were formed during warm albeit dry conditions (MIS 3.3). The star presents a significant change in shannon index after long-term incubation.

**SI Figure 2.** Accumulative CO_2_ production (nmol CO_2_ gdw^-1^) over time (days).

**SI Figure 3.** Methane production in rates (nmol CH_4_ gdw^-1^ d^-1^). The two panels indicate the samples with active methanogenesis ; A) MIS 3.1, and B) MIS 5e. Methane production after a threshold of 0.05 µmol CH_4_ g^-1^ is presented (according to Knoblauch et al., 2018). The sample K_1362.1, K _1362.1, K_1364.1 were flushed with N_2_ gas at day 1308. The sample L_0404.1, L _0404.2, L_0407.1 and L_0407.2 were flushed with N_2_ at day 732.

**SI Figure 4**. CH_4_ and CO_2_ production (nmol CO_2_ gdw^-1^) over time (days) in samples with active methanogenesis (MIS 3.1 and MIS 5e) (note the different y-axis scaling).

**SI Figure 5.** Number of reads versus number of Amplicon Sequence Variants (ASVs).

**SI Figure 6.** Rarefaction curve showing sample size versus number of species.

**SI Figure 7.** Presents all replicates of the initial samples. The lowest taxonomic assignment is present down to genus level. The samples are shown on the x-axis for all graphs, with samples from Bol’shoy Lyahovsky Island marked with “L” and those from the Kurungankh Island marked with “K. The blue fill color indicates samples that were formed under colder and drier and conditions in Figures A and B. The orange fill color indicates samples which was formed during warm albeit dry conditions. The red fill color indicates samples that were formed under periods with higher temperatures and rates of precipitation.

**SI Figure 8.** Krona charts of controls. A) Positive control with the methanogenic archaeon *Methanosarcina soligelidi SMA-21* as template. The sample contained of 25993 reads with 100% assigned to *Methanosarcina.* B and C are both negative controls with PCR water as template. B) This sample originate from a DNA extraction by Fastspin (MP Biomedicals) and it the contained 9 reads with 100% unassigned reads. C) This sample originate from a DNA extraction by Roboklon (EurX) and it contained 1 unassigned read.

**SI Table 1.** Original core ID, replicates and original sample ID of all samples included.

**SI Table 2.** Methane production over time (nmol CH_4_ gdw^-1^). The two schemes the samples with active methanogenesis ; A) MIS 3.1, and B) MIS 5e. Methane production after a threshold of 0.05 µmol CH_4_ g^-1^ is presented (according to Knoblauch et al., 2018).

**SI Table 3-7.** Includes for all initial and long-term incubated samples; Marine Isotope stage (MIS), archaeal reads per sample, number of archaeal ASVs, number of bacterial ASVs, Unassigned.

**SI Table 8a.** Correlation between Total organic carbon (TOC %) and Nitrogen (%), and the maximum CO_2_ and CH_4_ for each deposit respectively described according to Pearson’s correlation coefficient.

**SI Table 8b.** P-values for the significance of the Pearson correlation.

**SI Table 9:** One way Permanova with Bonferroni corrected p-values. The samples are grouped according to paleoenvironment. Samples from MIS 3.3 (L_0210) are grouped together with samples from cold and dry conditions (MIS 4). Samples are furthermore separated in initial and long-term incubated sample material (LT).
