# Supplementary material for: Methanogenic response to long-term permafrost thaw is determined by paleoenvironment
Source: FEMS Microbiol Ecol. 2020 Feb 7;96(3):fiaa021. doi: 10.1093/femsec/fiaa021 (PMC7046019; doi:10.1093/femsec/fiaa021)
Supplement: fiaa021_Supplemental_Files [file fiaa021_supplemental_files.zip › SI_Tables.docx]

**SI Table 1.** Original core ID, replicates and original sample ID of all samples included.

| **Age** | **Regional** | **Time interval** | **MIS** | **Core ID** | **Replicate** | **Sample ID** | **Site** | **Drilling coordinates** |
| --- | --- | --- | --- | --- | --- | --- | --- | --- |
| **Epoch** | **chrono-** |  |  |  |  |  |  |  |
|  | **stratigraphy** |  |  |  |  |  |  |  |
| **Holocene** | Holocene | Interglacial | **1**  <11 ka BP | L14_0502 | 1 2 | L14_0502.4 L14_0502.6 | Bol'shoy Lyakhovsky Island | 73.34996◦N 141.24156◦E |
| **Pleistocene** | Sartan | Glacial | **2**  14–29 ka BP | K09_1356 | 1 2 | K09_1356.4 K09_1356.6 | Kurungnakh Island | 72.333◦N 126.283◦E |
|  |  | (Stadial) |  | K09_1358 | 1 2 | K09_1358.4 K09_1358.5 |  |  |
|  |  |  |  | K09_1359 | 1 2 | K09_1359.4 K09_1359.5 |  |  |
|  |  |  |  |  |  |  |  |  |
|  | Kargin | Glacial (Interstadial) | **3**  34–420 ka BP |  |  |  |  |  |
|  |  |  | **3.1** | K09_1362 | 1 2 | K09_1362.4 K09_1362.6 |  |  |
|  |  |  |  | K09_1363 | 1 2 | K09_1363.4 |  |  |
|  |  |  |  | K09_1364 | 1 2 | K09_1364.4 K09_1364.5 |  |  |
|  |  |  |  |  |  |  |  |  |
|  |  |  |  |  |  |  |  |  |
|  |  | Glacial (Interstadial) |  | L14_0508 | 1 2 | L14_0508.4 L14_0508.6 | Bol'shoy Lyakhovsky Island | 73.34996◦N 141.24156◦E |
|  |  |  |  |  |  |  |  |  |
|  |  | Glacial (Interstadial) | **3.2** | L14_0203 | 1 2 | L14_0203.4 L14_0203.2 |  | 73.33623◦N 141.32761◦E |
|  |  | Glacial (Interstadial) | **3.3** | L14_0210 | 1 2 | L14_0210.4 L14_0210.6 |  |  |
|  | Zyryan | Glacial (Stadial) | **4**  40-52 ka BP | L14_0315 | 1 2 | L14_0315.4 L14_0315.6 |  | 73.33464◦N 141.32822◦E |
|  | Eemian Kazansevo | Interglacial | **5e**  ~136 ka BP | L14_0404 | 1 2 | L14_0404.4 L14_0404.6 |  | 73.34100◦N 141.28587◦E |
|  |  |  |  | L14_0405 | 1 2 | L14_0405.4 L14_0405.5 |  |  |
|  |  |  |  | L14_0407 | 1 2 | L14_0407.4 L14_0407.6 |  |  |

**SI Table 2.** Methane production over time (nmol CH_4_ gdw^-1^). The two schemes the samples with active methanogenesis ; A) MIS 3.1, and B) MIS 5e. Methane production after a threshold of 0.05 µmol CH_4_ g^-1^ is presented (according to Knoblauch et al., 2018).

| **A) MIS 3.1 K_1362.1** | **Incubation (days)** | **CH_4_ production (nmol gdw^-1^)** |  | **Incubation (days)** | **CH_4_** |  | **Incubation (days)** | **CH_4_** |
| --- | --- | --- | --- | --- | --- | --- | --- | --- |
|  |  |  |  |  |  |  |  |  |
| **K_1362.1** |  |  | **K_1363.1** | 970.9319 | 100.1 | **K_1364.1** |  |  |
|  |  |  |  | 1059.012 | 186.1 |  |  |  |
|  | 1133.08264 | 61.8 |  | 1133.075 | 273.6 |  |  |  |
|  | 1202.04097 | 77.6 |  | 1202.033 | 360.5 |  |  |  |
|  | 1247.13611 | 83.7 |  | 1247.129 | 411.0 |  |  |  |
|  | 1274.08542 | 89.4 |  | 1274.101 | 451.6 |  |  |  |
|  | 1308.16667 | 90.4 |  | 1308.16 | 454.0 |  |  |  |
|  | 2395 | 2790.0 |  | 2395.138 | 27701.2 |  | 2395.1347 | 1147.4 |
|  | 2501.2125 | 2790.0 |  | 2501.195 | 27703.7 |  | 2501.1833 | 1147.7 |

| **B) MIS 5e** | **Incubation (days)** | **CH_4_ production (nmol gdw^-1^)** | |  | **Incubation (days)** | **CH_4_** | |  | | **Incubation (days)** | | **CH_4_** |
| --- | --- | --- | --- | --- | --- | --- | --- | --- | --- | --- | --- | --- |
|  |  |  |  |  |  |  |  |  |  |  |  |  |
| **L_0404.1** | 230 |  | | **L_0404.2** |  |  | | **L_0405.1** | | 230 | | 68.5 |
|  | 284 |  | |  |  |  | |  | | 284 | | 81.2 |
|  | 387 | 92.6 | |  |  |  | |  | | 387 | | 143.3 |
|  | 444 | 308.0 | |  |  |  | |  | | 444 | | 153.6 |
|  | 786 | 1220.0 | |  | 786 | 327.0 | |  | | 786 | | 241.8 |
|  | 885 | 1740.0 | |  | 885 | 646.0 | |  | | 885 | | 292.8 |
|  |  |  | |  |  |  | |  | |  | |  |
| **B) MIS 5e** | **Incubation (days)** | | **CH_4_ production (nmol gdw^-1^)** |  | **Incubation (days)** | | **CH_4_** |  |  | |  |  |
|  |  |  |  |  |  |  |  |  |  | |  |  |
| **L_0405.2** | 230 | |  | **L_0407.2** | 230 | | 111.8 |  |  | |  |  |
|  | 284 | | 71.0 |  | 284 | | 168.7 |  |  | |  |  |
|  | 387 | | 71.5 |  | 387 | | 259.3 |  |  | |  |  |
|  | 444 | | 84.2 |  | 444 | | 396.8 |  |  | |  |  |
|  | 786 | | 133.0 |  | 786 | | 2481.2 |  |  | |  |  |
|  | 1229 | | 279.0 |  | 885 | | 3359.6 |  |  | |  |  |

**SI Table 3-7.** Includes for all initial and long-term incubated samples; Marine Isotope stage (MIS), archaeal reads per sample, number of archaeal ASVs, number of bacterial ASVs, Unassigned.

**SI Table 3.**

| **Initial samples** | **L_0502** | **L_0502** | **K_1356** | **K_1356** | **K_1358** | | **K_1358** | **K_1359** | | **K_1359** | **K_1362** | **K_1362** | **K_1363** | **K_1363** |
| --- | --- | --- | --- | --- | --- | --- | --- | --- | --- | --- | --- | --- | --- | --- |
|  |  |  |  |  |  |  |  |  |  |  |  |  |  |  |
| Replicate | **1** | **2** | **1** | **2** | **1** | | **2** | **1** | | **2** | **1** | **2** | **1** | **2** |
| **Marine Isotope Stage (MIS)** | 1 | 1 | 2 | 2 | 2 | | 2 | 2 | | 2 | 3.1 | 3.1 | 3.1 | 3.1 |
| **Archaeal reads** | 262655 | 548 | 156551 | 129430 | 45042 | 5545 | | 5058 | | 108925 | 21785 | 58630 | 25289 | 74879 |
| **Number of archaeal ASVs** | 36 | 9 | 55 | 64 | 10 | 5 | | 18 | 46 | | 38 | 67 | 34 | 40 |
| **Bacterial ASVs** | 0 | 0 | 0 | 225 | 0 | 0 | | 229 | 2 | | 0 | 72 | 0 | 0 |
| **Unassigned** | 0 | 0 | 0 | 0 | 0 | 0 | | 0 | 0 | | 0 | 0 | 0 | 0 |
|  |  |  |  |  |  |  | |  |  | |  |  |  |  |

**SI Table 4.**

| **Initial samples** | **K_1364** | **K_1364** | **L_0508** | **L_0508** | **L_0203** | **L_0203** | **L_0210i** | **L_0404** | **L_0404i** | **L_0407i** | **L_0407i** |
| --- | --- | --- | --- | --- | --- | --- | --- | --- | --- | --- | --- |
|  |  |  |  |  |  |  |  |  |  |  |  |
| Replicate | 1 | 2 | 1 | 2 | 1 | 2 | 1 | 2 | 1 | 2 | 1 |
| **Marine Isotope Stage (MIS)** | 3.1 | 3.1 | 3.1 | 3.1 | 3.2 | 3.2 | 3.3 | 5e | 5e | 5e | 5e |
| **Archaeal reads** | 1309 | 12386 | 164269 | 210634 | 461075 | 406693 | 36502 | 360486 | 425087 | 273178 | 164599 |
| **Number of archaeal ASVs** | 11 | 3 | 20 | 31 | 45 | 53 | 9 | 98 | 91 | 25 | 31 |
| **Bacterial ASVs** | 1595 | 0 | 0 | 0 | 57 | 144 | 0 | 0 | 0 | 0 | 0 |
| **Unassigned** | 0 | 0 | 0 | 0 | 0 | 0 | 0 | 0 | 0 | 0 | 0 |

**SI Table 5.**

| **Long term incubated samples** | **L_0502** | **L_0502** | **K_1356** | **K_1356** | **K_1358** | **K_1358** | **K_1359** | **K_1359** | **K_1362** | **K_1362** | **K_1363** | **K_1363** |
| --- | --- | --- | --- | --- | --- | --- | --- | --- | --- | --- | --- | --- |
| **Replicate** | 1 | 2 | 1 | 2 | 1 | 2 | 1 | 2 | 1 | 2 | 1 | 2 |
| **Marine Isotope Stage (MIS)** | 1 | 1 | 2 | 2 | 2 | 2 | 2 | 2 | 3.1 | 3.1 | 3.1 | 3.1 |
| **Archaeal reads** | 443750 | 443750 | 58245 | 35841 | 59901 | 51972 | 45779 | 71840 | 55926 | 85105 | 102777 | 221832 |
| **Number of archaeal ASVs** | 63 | 41 | 38 | 27 | 62 | 36 | 39 | 56 | 32 | 43 | 17 | 37 |
| **Bacterial ASVs** | 0 | 0 | 0 | 0 | 0 | 0 | 0 | 0 | 0 | 0 | 0 | 0 |
| **Unassigned** | 0 | 0 | 0 | 0 | 0 | 3 | 0 | 0 | 0 | 0 | 0 | 0 |

**SI Table 6.**

| **Long term incubated samples** | **K_1364** | **K_1364** | **K_1364** | **L_0508** | **L_0508** | **L_0203** | **L_0203** | **L_0210** | **L_0210** | **L_0404** | **L_0404** | **L_0404** |
| --- | --- | --- | --- | --- | --- | --- | --- | --- | --- | --- | --- | --- |
| **Replicate** | **1** | **1** | **2** | **1** | **2** | **1** | **2** | **1** | **2** | **1** | **2** | **1** |
| **Marine Isotope Stage (MIS)** | 3.1 | 3.1 | 3.1 | 3.1 | 3.1 | 3.2 | 3.2 | 3.3 | 3.3 | 5e | 5e | 5e |
| **Archaeal reads** | 18572 | 29476 | 339272 | 12896 | 45191 | 3575 | 627561 | 621680 | 353404 | 13445 | 78619 | 556518 |
| **Number of archaeal ASVs** | 7 | 1 | 187 | 8 | 8 | 18 | 20 | 14 | 9 | 9 | 21 | 46 |
| **Bacterial ASVs** | 0 | 0 | 815 | 0 | 0 | 19515 | 114 | 0 | 0 | 0 | 0 | 0 |
| **Unassigned** | 0 | 0 | 40 | 0 | 0 | 2187 | 0 | 0 | 0 | 0 | 0 | 0 |

**SI Table 7.**

| **Long term incubated samples** | **L_0405** | **L_0405** | **L_0407** | **L_0407** |
| --- | --- | --- | --- | --- |
| **Replicate** | 1 | 2 | 1 | 2 |
| **Marine Isotope Stage (MIS)** | 5e | 5e | 5e | 5e |
| **Archaeal reads** | 44251 | 106799 | 459358 | 402745 |
| **Number of archaeal ASVs** | 11 | 18 | 19 | 36 |
| **Bacterial ASVs** | 0 | 0 | 0 | 0 |
| **Unassigned** | 0 | 0 | 0 | 0 |

**SI Table 8a. Correlation between Total organic carbon (TOC %) and Nitrogen (%), and the maximum CO_2_ and CH_4_ for each deposit respectively described according to Pearson’s correlation coefficient.**

| **Pearson correlation (r-values)** | **Max CO_2_** | **Max CH_4_** |
| --- | --- | --- |
| **TOC (%)** | **0.648681376** | -0.219593084 |
| **N (%)** | **0.793136431** | 0.006470838 |

**SI Table 8b. P-values for the significance of the Pearson correlation.**

| **Pearson correlation**  **(p-values)** | **Max CO_2_** | **Max CH_4_** |
| --- | --- | --- |
| TOC (%) | ***0.001** | 0.24 |
| N (%) | ***0.0009** | 0.97 |

**SI Table 9:** One way Permanova with Bonferroni corrected p-values. The samples are grouped according to paleoenvironment. Samples from MIS 3.3 (L_0210) are grouped together with samples from cold and dry conditions (MIS 4). Samples are furthermore separated in initial and long-term incubated sample material (LT).

| **One way permanova** |  | **Initial** | **Initial** | **LT** | **LT** |
| --- | --- | --- | --- | --- | --- |
|  |  | **Warm** | **Cold** | **Warm** | **Cold** |
| **Initial** | **Warm** |  | 1 | 1 | 0.1476 |
| **Initial** | **Cold** | 1 |  | 0.0042 | 1 |
| **LT** | **Warm** | 1 | 0.0042 |  | 0.0006 |
| **LT** | **Cold** | 0.1476 | 1 | ***0.0006** |  |
