# Supplementary figures and images for: Methanogenic response to long-term permafrost thaw is determined by paleoenvironment
Source: FEMS Microbiol Ecol. 2020 Feb 7;96(3):fiaa021. doi: 10.1093/femsec/fiaa021 (PMC7046019; doi:10.1093/femsec/fiaa021)

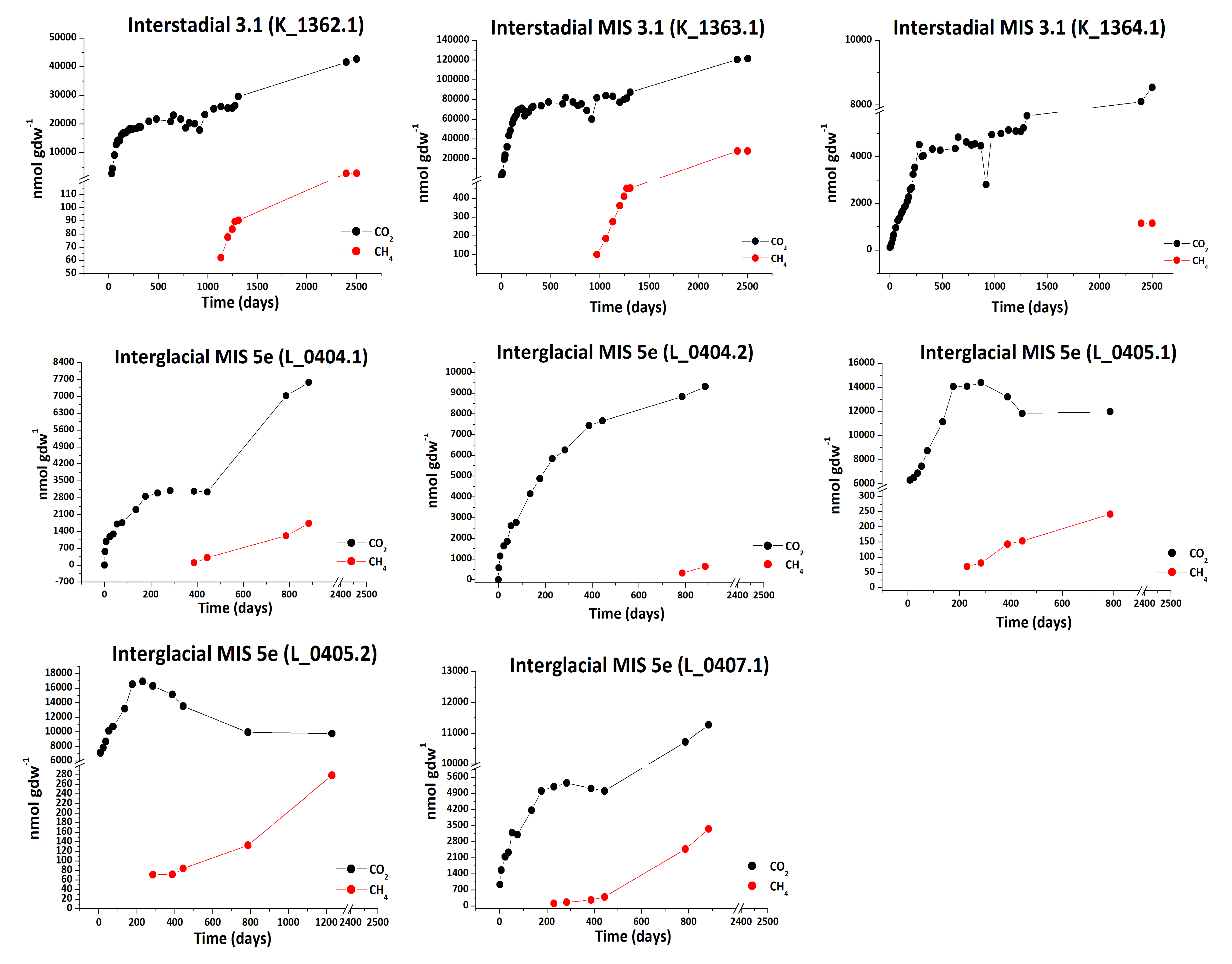

Supplement: fiaa021_Supplemental_Files [file fiaa021_supplemental_files.zip › Figure_SI_4_600dpi_TIFF.tif]

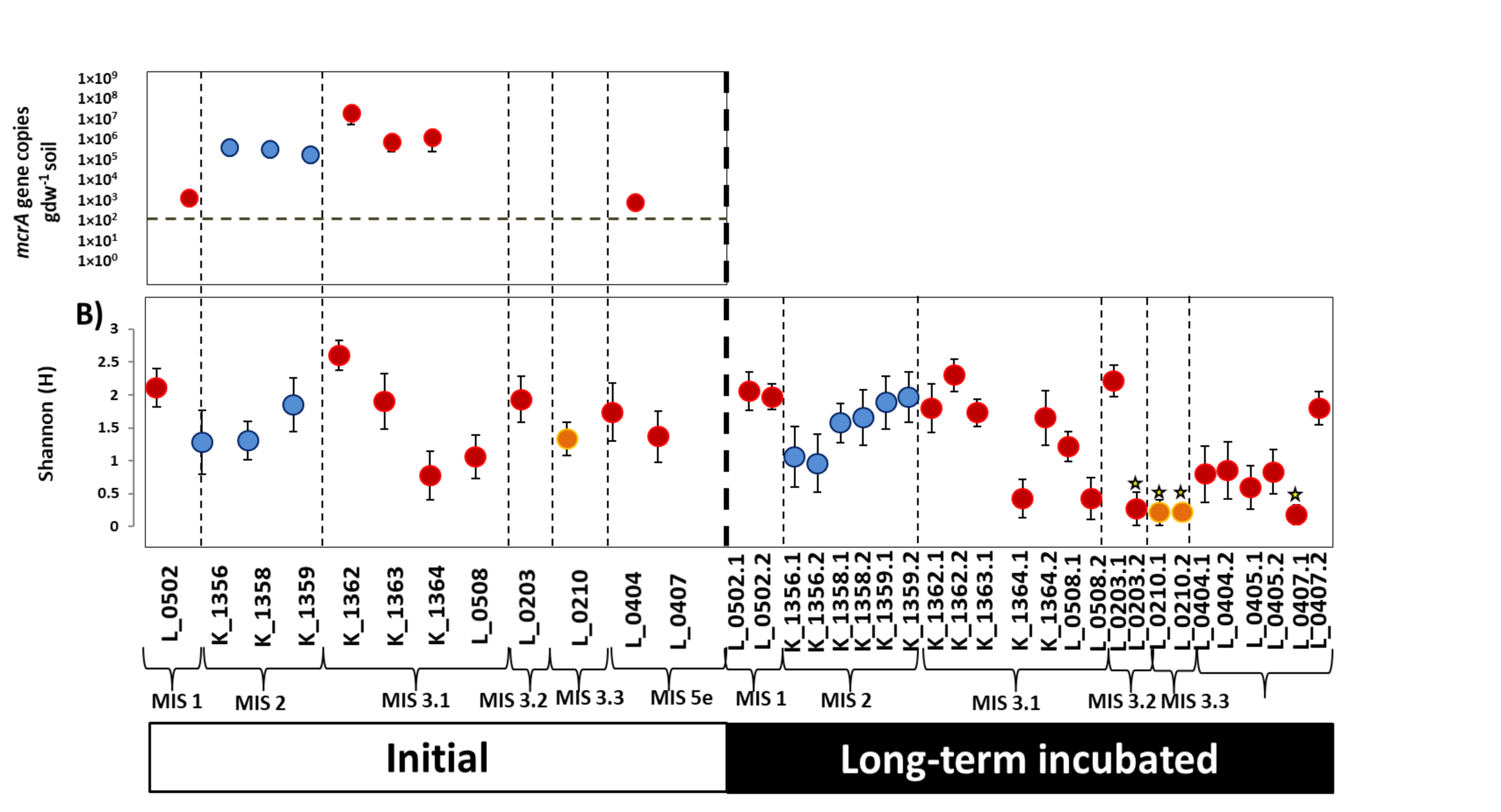

Supplement: fiaa021_Supplemental_Files [file fiaa021_supplemental_files.zip › SI_Figure 1_600dpi_TIFF.tif]

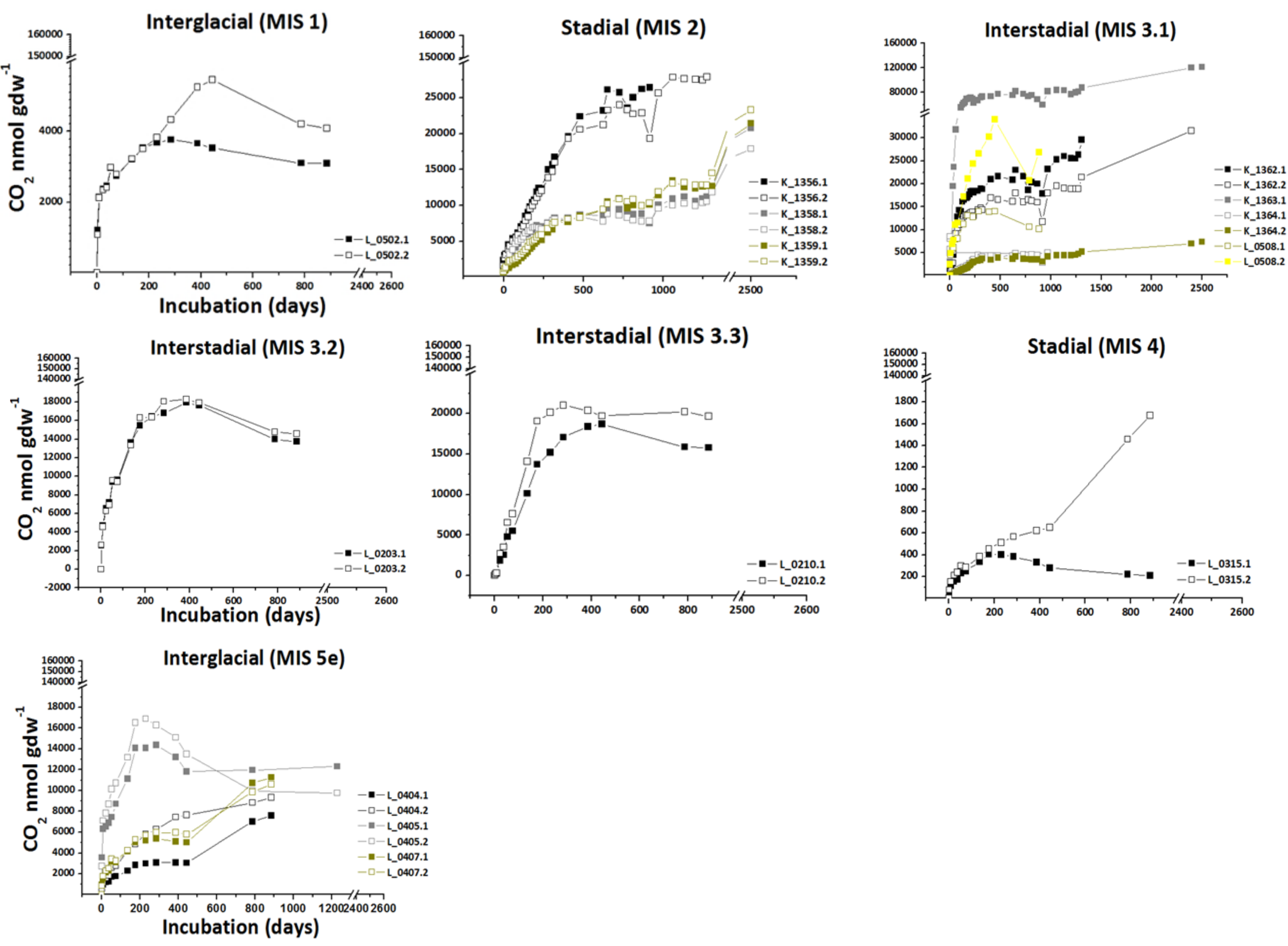

Supplement: fiaa021_Supplemental_Files [file fiaa021_supplemental_files.zip › SI_Figure_2_600dpi_TIFF.tif]

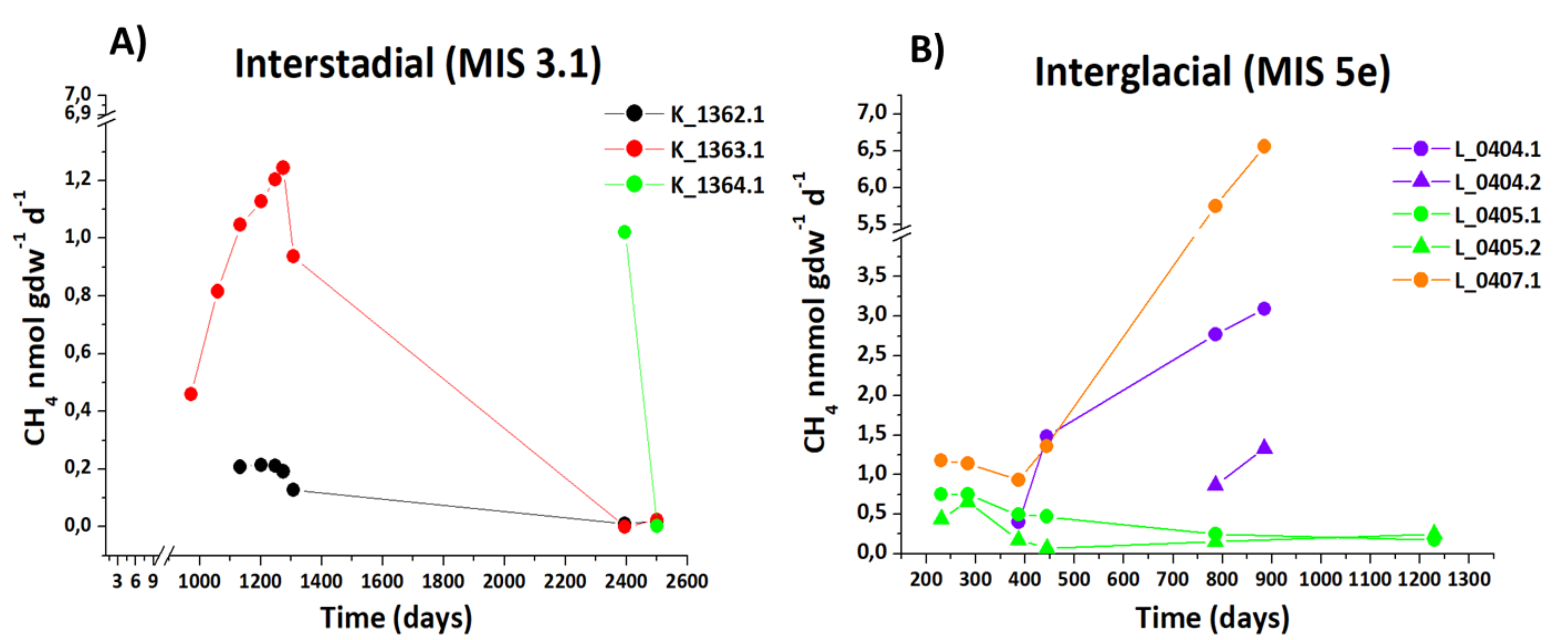

Supplement: fiaa021_Supplemental_Files [file fiaa021_supplemental_files.zip › SI_Figure_3_600dpi_TIFF.tif]

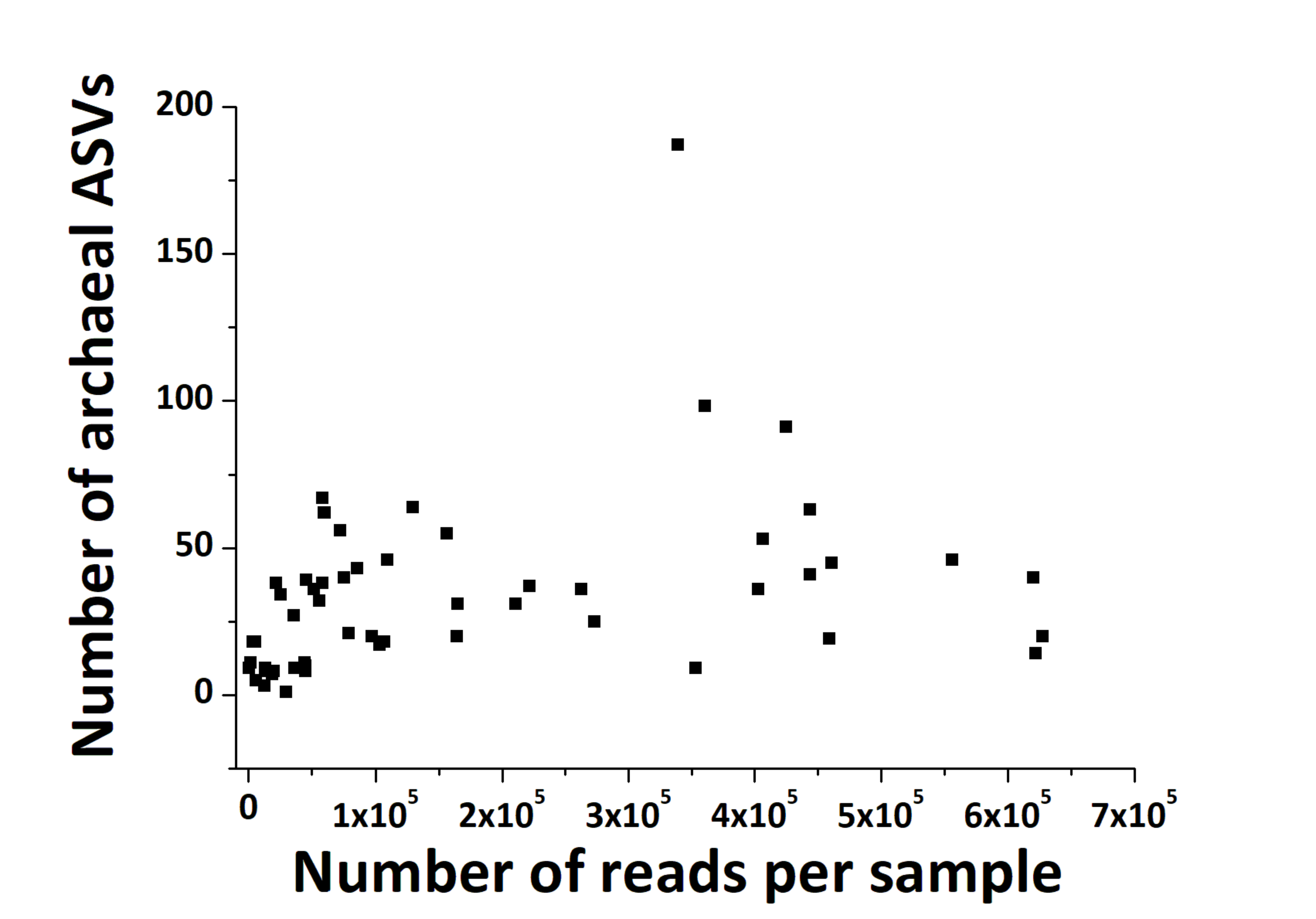

Supplement: fiaa021_Supplemental_Files [file fiaa021_supplemental_files.zip › SI_Figure_5_600dpi_TIFF.tif]

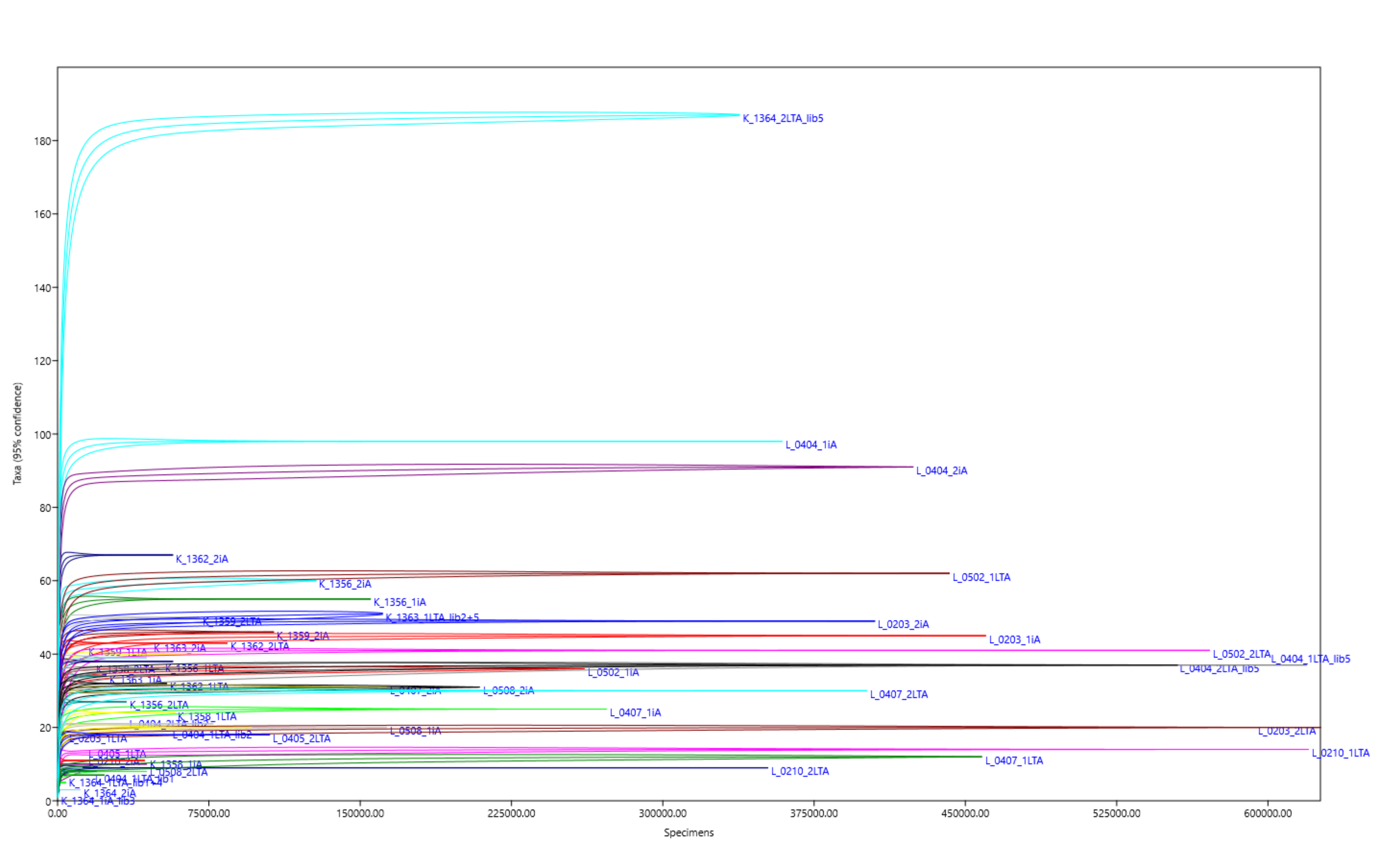

Supplement: fiaa021_Supplemental_Files [file fiaa021_supplemental_files.zip › SI_Figure_6_600dpi_TIFF.tif]

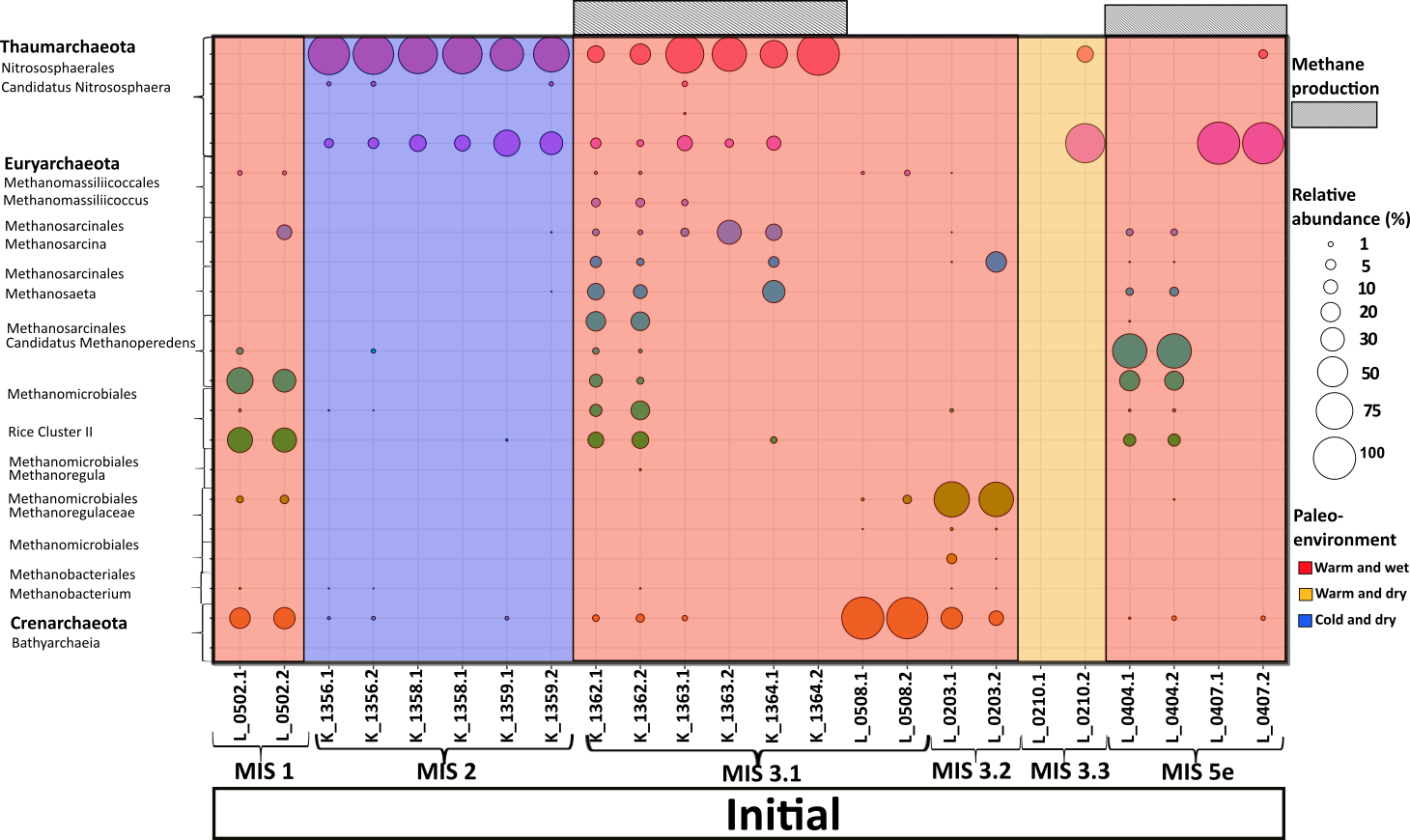

Supplement: fiaa021_Supplemental_Files [file fiaa021_supplemental_files.zip › SI_Figure_7_600dpi_TIFF.tif]

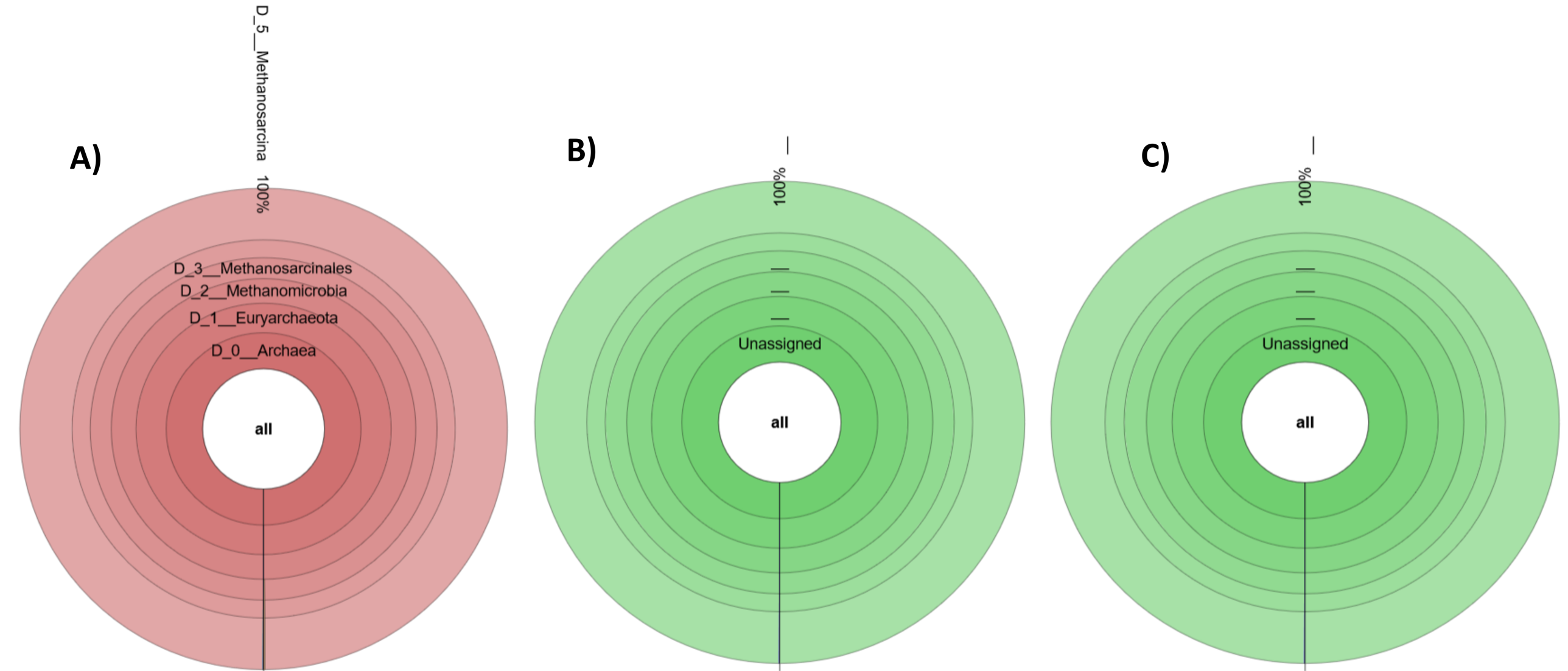

Supplement: fiaa021_Supplemental_Files [file fiaa021_supplemental_files.zip › SI_Figure_8_600dpi_TIFF.tif]
